# Expression of smooth muscle-like effectors and core cardiomyocyte regulators in the contractile papillae of *Ciona*

**DOI:** 10.1186/s13227-020-00162-x

**Published:** 2020-08-03

**Authors:** Christopher J. Johnson, Florian Razy-Krajka, Alberto Stolfi

**Affiliations:** Georgia Institute of Technology, Atlanta, GA USA

## Abstract

**Background:**

The evolution of vertebrate smooth muscles is obscured by lack of identifiable smooth muscle-like cells in tunicates, the invertebrates most closely related to vertebrates. A recent evolutionary model was proposed in which smooth muscles arose before the last bilaterian common ancestor, and were later diversified, secondarily lost or modified in the branches leading to extant animal taxa. However, there is currently no data from tunicates to support this scenario.

**Methods and results:**

Here, we show that the axial columnar cells, a unique cell type in the adhesive larval papillae of the tunicate *Ciona,* are enriched for orthologs of vertebrate smooth/non-muscle-specific effectors of contractility, in addition to developing from progenitors that express conserved cardiomyocyte regulatory factors. We show that these cells contract during the retraction of the *Ciona* papillae during larval settlement and metamorphosis.

**Conclusions:**

We propose that the axial columnar cells of *Ciona* are a myoepithelial cell type required for transducing external stimuli into mechanical forces that aid in the attachment of the motile larva to its final substrate. Furthermore, they share developmental and functional features with vertebrate myoepithelial cells, vascular smooth muscle cells, and cardiomyocytes. We discuss these findings in the context of the proposed models of vertebrate smooth muscle and cardiomyocyte evolution.

## Background

The evolutionary history of the various muscle types found in animals remains unresolved [80, 95, 96]. In vertebrates, muscles are classified into 3 major types according to their structure and functions, not taking into account their developmental or evolutionary origins: smooth muscles, cardiac striated muscles (composed of cells known as cardiomyocytes), and non-cardiac striated muscles, the latter being mostly skeletal muscles [95]. Vertebrate smooth muscles are those muscles that lack repeated contractile actin–myosin units [44, 45] and are primarily defined by smooth muscle-specific effectors of contractility whose regulation is independent of the myogenic regulatory factors (MRFs: in humans MYOD1, MYOG, MYF5, and MYF6) that specify striated muscles [6, 96]. Myoepithelial cells are smooth muscle-like cells that are arranged as epithelia and are associated with vertebrate secretory glands and the iris dilator muscle [62]. Most myoepithelial cells are derived from surface ectoderm, non-migratory neurectoderm, and even endoderm [3, 29, 50], as opposed to the predominantly mesodermal or neural crest origin of conventional smooth muscles [28, 78]. While they share all of their contractile apparatus with smooth muscles [25, 63], little is known about the regulation of their developmental and evolutionary trajectories [59, 68].

Phylogenomic analyses indicate that all bilaterians have striated muscles that likely evolved in their last common ancestor, while cnidarians evolved striated muscles independently [96]. In contrast, the particular smooth muscles found in vertebrates, mainly visceral and vascular, have long been thought to represent vertebrate innovations for several reasons. First, smooth muscles are absent from the major invertebrate model organism *Drosophila* [106]. Second, effector proteins found in vertebrate smooth muscles and myoepithelia [32] are usually encoded by vertebrate-specific gene duplications and are distinct from those operating in non-muscles cells and in a majority of striated muscles. These include smooth muscle-specific actin and myosins [38, 86, 96], calponin [103], and myosin light chain kinase (encoded by *Mylk,* though also expressed in non-muscle cells) [54]. Third, smooth muscles are also thought to be absent from tunicates [14, 97], the sister group to the vertebrates within the phylum Chordata [24]. Although tunicate adult body wall muscles are structurally non-striated, they use conventional striated muscle contractility effectors and are specified by MRF, suggesting they have secondarily lost their striations [51, 80]. Recent studies have revealed that tunicates possess homologs of various structures, cell types, and tissues that were previously presented as vertebrate novelties [1, 2, 27, 98, 100]. Since these innovations likely predate the emergence of vertebrates, such studies have helped shape our models of chordate evolution [89].

Recently, it was proposed that vertebrate smooth muscles are homologous to visceral smooth muscles of the marine annelid *Platynereis dumerilii* [8], and that striated cardiomyocytes evolved from an ancestral smooth muscle-like cell independently in various clades including arthropods and vertebrates. This model is based on the fact that *P. dumerilii* visceral smooth muscles express homologs of vertebrate smooth muscle and cardiomyocyte regulators, and assumes visceral smooth muscles were secondarily lost from arthropods and nematodes [8, 38]. Therefore, identifying and characterizing potential smooth muscle homologs in tunicates, the sister group to the vertebrates, is paramount to further resolving the evolutionary origins and diversification of muscle types. Are the different smooth muscle subtypes of vertebrates recent innovations, or do they predate the subfunctionalization of non-muscle/smooth muscle effectors that occurred after vertebrates diverged from tunicates? Answering such questions is key to understanding overall human evolutionary history, since muscles such as those of the “new head” and the multi-chambered heart are thought to have played an outsized role in the evolution of vertebrates [34, 36].

**Fig. 1:**
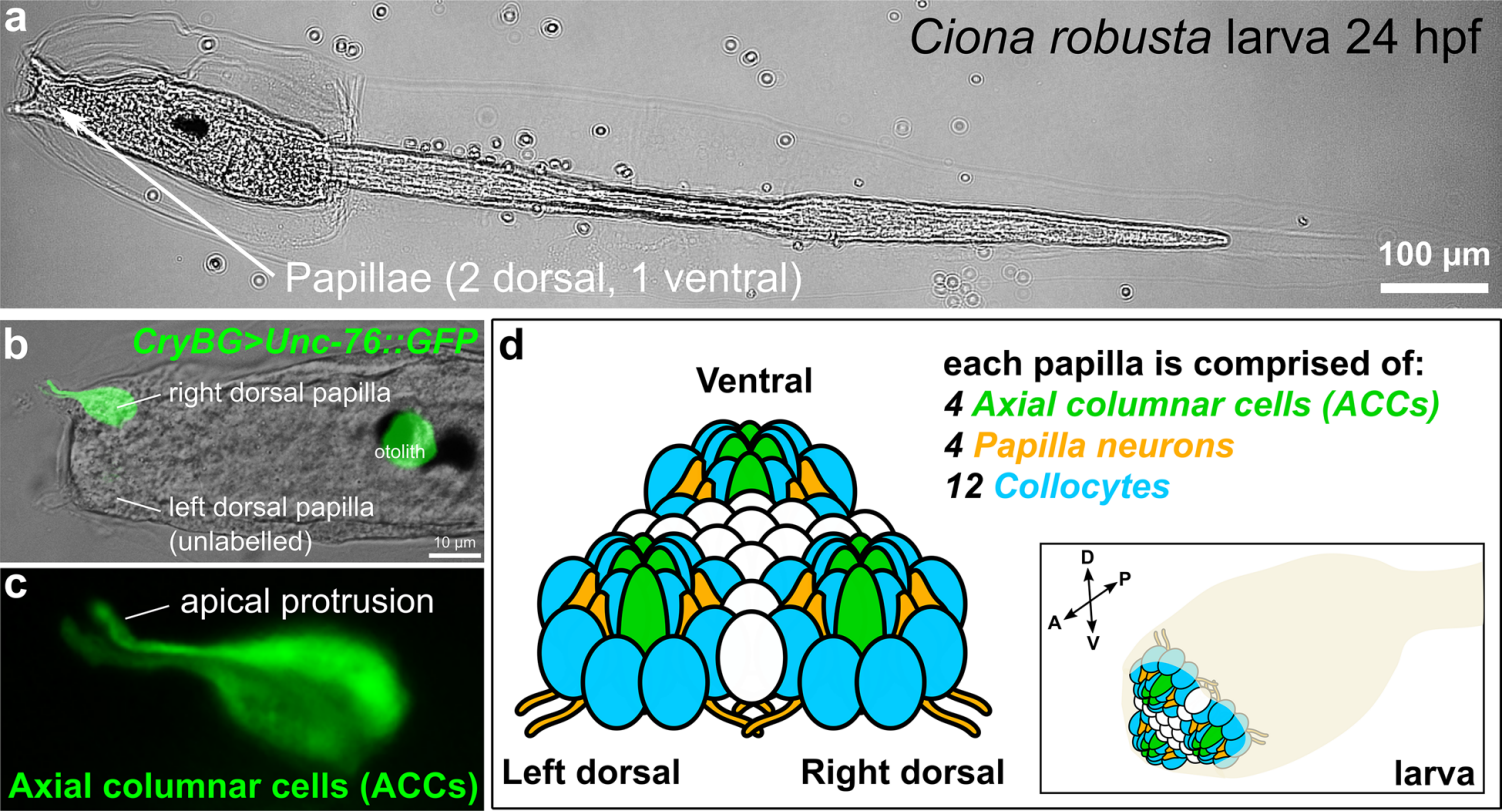
The papillae of *Ciona* larvae. **a** A *Ciona robusta (intestinalis Type A)* larva, showing the 3 papillae. **b** Larva electroporated with the reporter plasmid *CryBG *> *Unc*-*76::GFP* (Cirobu.REG.KhS605. 16,789–17,833, Shimeld et al., which labels axial columnar cells (ACCs) and unrelated otolith cell. This larva is a transgenic mosaic, so the left dorsal papilla is unlabeled. The ventral papilla is also unlabeled but out of focus. **c** Magnified view of labeled papilla in **b**, without brightfield overlay, showing ACCs labeled by *CryBG *> *Unc*-*76::GFP* expression. Each has a characteristic apical, finger-like protrusion that extends through a fenestration of the larval cellulose tunic around the apical tip of the papilla. **d** Cartoon diagram of the major cell types of the three papillae and their approximate arrangement in the larva. Cell types are identified by color code and described in reference [121]. Cells of unknown number and type in between the three papillae are represented in white. Sizes not to scale

Recently, we used single-cell RNAseq (scRNAseq) to profile cells isolated from larval brains of the model tunicate *Ciona robusta (intestinalis type A)* [91], which unexpectedly revealed the transcriptome of a small group of transcriptionally distinct cells that comprised mostly axial columnar cells (ACCs) [75, 121] of the papillae. The papillae (Fig. 1a), also known as “palps”, are a set of three anterior organs that regulate larval settlement and metamorphosis [43, 70]. The tadpole-type larvae of *Ciona* spend a short time swimming in search for a hard substrate on which to settle and metamorphose into the sessile juvenile/adult form, and this choice is mediated by the sensory and adhesive functions of the papillae in response to unknown biotic and abiotic cues [18, 39, 43, 55, 70, 82, 102, 108]. Although the ACCs of *Ciona* have been variably called “Papilla/Palp Neurons” [47, 85, 91], “Papilla/Palp Sensory Cells” [42], here we report that their transcriptional profile suggests that they do not closely resemble neurons but rather that they appear to possess contractile properties, which drives the retraction of the papillae, a feature that appears to be conserved in other tunicate species and that may be important for the attachment of the larva to hard substrates during settlement and metamorphosis. We show that the orthologs of vertebrate smooth muscle/myoepithelial cell effectors and conserved cardiomyocyte transcription factors are expressed at various time points in the precursor cells that give rise to the papillae, including the ACCs. We discuss these findings in the context of smooth muscle evolution and the smooth-to-striated model of chordate cardiomyocyte evolution.

## Methods

### *Ciona robusta* collection and handling

*Ciona robusta* (intestinalis Type A) were collected in San Diego, CA (M-REP). Embryos were fertilized, dechorionated, and electroporated (10–70 µg of plasmid per 700 µl of solution) as previously described [15, 16]. Sequences of previously unpublished plasmids and in situ hybridization probe templates are included in Additional file 1. Fluorescent whole-mount in situ hybridizations were carried out as described [5, 46], with incubation with 0.5 µg/ml of proteinase K for larval stages and 1 µg/ml for tailbud stages. Embryos and larvae were imaged using a Leica inverted DMi8 or DMIL LED epifluorescence compound microscope.

### Single-cell RNAseq re-clustering

**Fig. 2:**
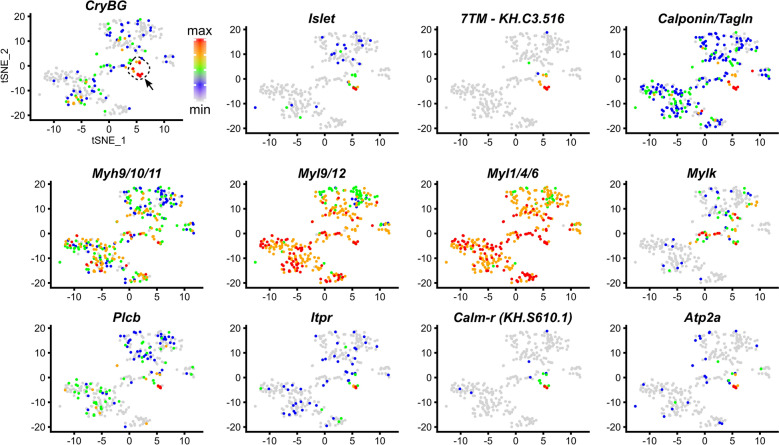
Single-cell RNAseq differential gene expression “maps” of ACC markers. tSNE plots of re-clustered larval cells from Sharma et al. [91]. ACC-containing subcluster (arrow pointing to red- and orange-colored cells) is marked by high expression of *Beta/gamma Crystallin (CryBG),* and *Islet,* based on previous findings (see text for detail). Cells colored by relative gene expression, normalized as maximum (red) to minimum (grey) values for each gene, as indicated by color scale at top left. See also Additional file 1: Fig. S1

Processed scRNAseq data previously published [91] was re-analyzed using Seurat v3 [88, 101], first by clustering all cells using PCA and tSNE, followed by identification of the cell cluster containing the ACCs by selecting the one (Cluster 2) with cells showing highest relative expression of *β/γ Crystallin* [92]. Cluster 2 was re-clustered by reiterating the same method, and expression distribution for each candidate gene was plotted against this re-clustering (Fig. 2). The code for this is available at https://osf.io/5dc4u/.

### GCamp6s imaging and quantification

Embryos electroporated with 30 µg of *CryBG*>*GCamp6s* plasmid per 700 µl of solution were placed in a coverslip-bottom dish and imaged live using a Leica DMi8 inverted compound microscope equipped with a Leica DFC3000 G monochrome CCD camera. For each frame of a single larva imaged, fluorescence intensity values in a particular region of interest (ROIs, Fig. 5b) were averaged. ROI 1 was the main one, encompassing the apical protrusions of the ACCs, while ROI 2 was a negative control region outside the ACCs, to get a sense of background fluorescence variation. ΔF/F0 values were calculated by subtracting the intensity value in the initial frame from each frame’s intensity value, then normalized to the maximal value.

## Results

### ACCs express the orthologs of vertebrate smooth muscle genes

Our scRNAseq analysis of the *C. robusta* larval brain dissociated at 20 h post-fertilization, 20 °C [91], previously revealed a small group of 12 cells that were identified as comprising primarily ACCs likely mixed with other papilla cell types, on the basis of their expression of specific markers such as *β/γ Crystallin (CryBG,* unique ID *Cirobu.g00014792)* [92]. Although that study meant to target specifically brain cells, cells from the papillae were unintentionally isolated due to low-level expression of the fluorescent reporter (*Fascin *> *tagRFP)* that was used to collect the cells by fluorescence-activated cell sorting. In *Ciona,* exactly four *CryBG *+ACCs are found at the center of each papilla (Fig. 1b, c), and are morphologically distinct from the other cell types of the papillae, which also include putative adhesive-secreting collocytes and sensory neurons [120, 121] (Fig. 1d). These cells were proposed [26, 75] to be homologous to the ACCs in the much larger, more complex papillae of several colonial species [9, 17, 35, 107, 110], but this is based solely on their position and shape, and not on molecular or functional criteria. Here we continue to use the term ACC, for the sake of consistency.

Some studies, including our own previous paper, have assumed the ACCs of *Ciona* to be neurons [42, 47, 85, 91], based on the report of axon-bearing central cells in the papillae of the species *Phallusia mammillata* [26, 94] and *Clavelina lepadiformis* [74]. However*, Ciona* ACCs have been consistently shown to lack axons or neurites of any kind [113, 121]. When we looked in detail at differential gene expression in our single-cell dataset, we were surprised to find that the ACC cluster was enriched for transcripts orthologous to several vertebrate smooth muscle markers (Table 1 and Additional file 2). In particular, these include transcripts encoding the *Ciona* orthologs of non-muscle/smooth muscle-specific myosin chains: *Myosin heavy chain 9/10/11* (*Myh9/10/11, Cirobu.g00002290*, LogFC = 1.2) and *Myosin light chain 9/12* (*Myl9/12, Cirobu.g00001952*, LogFC = 0.9). According to the ANISEED database of tunicate gene sequences [19], Myh9/10/11/14 shows greatest identity to human MYH10 (63%) and MYH11 (62%) proteins, which are vertebrate non-muscle and smooth muscle heavy chains, respectively [96]. Previous phylogenetic analysis showed this protein (originally called Ci-MHC1) groups with non-muscle/smooth muscle myosin heavy chains [14]. Likewise, Myl9/12 shows greatest similarity to vertebrate non-muscle/smooth muscle regulatory light chains MYL9, MYL12A, and MYL12B (all ~ 77% identity), and phylogenetically groups with said vertebrate non-muscle/smooth muscle proteins [14]. Finally, ACCs also showed enriched expression of the “essential” (“alkali”, or non-regulatory) *Myosin light chain 1/4/6* gene (*Myl1/4/6, Cirobu.g00000433*, LogFC = 1.0). Although this gene shows greater identity to fast skeletal MYL1 (64%) and cardiac/embryonic MYL4 (62%) than to non-muscle/smooth muscle MYL6 (59%), previous phylogenetic analyses suggested that tunicate and vertebrate essential light chain genes all arose from a single gene in the last common ancestor, undergoing independent duplications and subfunctionalization after the tunicate/vertebrate split [14].

**Table 1:** Selected genes with transcripts enriched in the axial columnar cells compared to neural cells

ANISEED ID	KH ID	Name	Top BLASTP hit in human	AveLog(FC)	*p* value	Adj. *p* value	Pct-1	Pct-2
*Cirobu.g00014792*	*KH.S605.3*	*Crystallin Beta/Gamma*	*CRYBB2*	5.9	3E−48	4.02E−44	1	0.25
*Cirobu.g00005701*	*KH.C3.516*	*7TM*-*KH.C3.516*	*None*	4.1	6E−22	8.18E−18	0.92	0
*Cirobu.g00006660*	*KH.C4.559*	*Calponin/Transgelin*	*CNN1*	3.5	5E−27	6.18E−23	1	0.16
*Cirobu.g00014804*	*KH.S610.1*	*Calmodulin*-*related*	*CALM2*	3.2	3E−17	3.68E−13	0.83	0.07
*Cirobu.g00010771*	*KH.L116.40*	*Atp2a (SERCA)*	*ATP2A2*	2.9	5E−21	7.19E−17	0.83	0.16
*Cirobu.g00000306*	*KH.C1.1276*	*Calmodulin*-*related*	*CALM1; CALM3; CALML3*	2.8	7E−15	9.26E−11	0.92	0.18
*Cirobu.g00009220*	*KH.C8.573*	*Calmodulin*-*related*	*CALM1; CALM2; CALM3*	2.3	3E−13	3.54E−09	0.83	0.19
*Cirobu.g00009924*	*KH.C9.384*	*Mylk (MLCK)*	*MYLK4*	2.2	1E−14	1.65E−10	0.92	0.04
*Cirobu.g00009359*	*KH.C8.70*	*IP3 Receptor*	*ITPR1*	2.1	2E−14	2.42E−10	0.83	0.2
*Cirobu.g00011396*	*KH.L152.2*	*Islet*	*ISL1*	1.9	8E−11	1.08E−06	0.83	0.08
*Cirobu.g00006733*	*KH.C4.626*	*Plec/Dsp/Eppk*	*PLEC; DSP; EPPK1*	1.8	4E−09	0.0000476	0.67	0.07
*Cirobu.g00004386*	*KH.C2.209*	*Calmodulin*-*related*	*CALM1; CALM3; CALML3*	1.5	8E−08	0.001067	0.75	0.17
*Cirobu.g00006467*	*KH.C4.381*	*PLC beta*	*PLCB2*	1.2	1E−04	1	0.67	0.15
*Cirobu.g00002290*	*KH.C11.456*	*Myh9/10/11*	*MYH10*	1.1	9E−06	0.117273	0.83	0.28
*Cirobu.g00000433*	*KH.C1.216*	*Myl1/4/6*	*MYL1*	1.0	7E−08	0.000904	1	0.93
*Cirobu.g00001952*	*KH.C11.143*	*Myl9/12*	*MYL9; MYL12A; MYL12B*	0.9	2E−06	0.023005	0.92	0.79

List of genes discussed in this paper, identified by unique gene ID (Cirobu.gxxxxxxxx), KyotoHoya (KH) gene model ID, name given in this study, and Top BLASTP (after translation) hit in humans. Single-cell RNAseq data from [91] using Seurat [88] include Average Log(FC), *p* value, adjusted *p* value, Pct-1, and Pct-2. See text or [88] for details about scRNAseq values. See Additional file 2 for full ACC scRNAseq gene list

Although vertebrate non-muscle and smooth muscle-specific myosins are encoded by subfunctionalized gene duplications, invertebrates like *P. dumerilii* have smooth muscles that utilize more ancestral-like, non-specialized non-muscle/smooth muscle myosins [8]. However, this also means that the mere expression of non-muscle/smooth muscle-like myosins in *Ciona* ACCs does not necessarily indicate that they represent a smooth muscle-like cell type. Yet we also found that ACCs were enriched for the expression of additional effector genes closely associated with smooth muscle cells in vertebrates. For instance, Calponin functions to inhibit actin-dependent myosin activity in vertebrate smooth muscles [67, 77], in a manner analogous to Troponin in striated muscle activity [103]. We found that the ACCs highly express the *Ciona* ortholog *Calponin/Transgelin* (*Cirobu.g00006660*, LogFC = 3.5). ACCs are also enriched for transcripts encoding an ortholog of vertebrate plectin/desmoplakin/epiplakin (*Cirobu.g00006733,* LogFC = 1.8), which shows the following identities to human proteins: 39% to plectin, 37% desmoplakin, 35% epiplakin. This protein family represents a major component of desmosomes in general, and dense plaques specifically in smooth muscle [105], which link actin and intermediate filaments at the plasma membrane and are important for contraction. Among its human orthologs, epiplakin is enriched in smooth muscle [111]. Finally, vertebrate smooth muscle contractions are regulated by calcium signaling via calmodulin-activated myosin light chain kinase (MLCK), which phosphorylates and activates myosin regulatory light chains [54]. We found that the ACCs also express *Myosin light chain kinase* (*Mylk, Cirobu.g00009924,* LogFC = 2.2), which encodes a protein with greatest identity to non-muscle/smooth muscle MLCK4 (52%) and MLCK (47%) in humans. In contrast, they are not enriched for genes encoding the major contractility effectors expressed in larval tail muscles, which closely resemble vertebrate striated muscles [14, 80].

Other effectors important for calcium-mediated activation of MLCK in vertebrate smooth muscles (reviewed in [87]) include calmodulin family members, which bind calcium and activate MLCK in a calcium-dependent manner [41]; IP3 receptors, which are activated by IP3 and release Ca2+ from intracellular stores for calmodulin activation [11]; phospholipase C, which cleaves PIP2 to produce IP3 as well as DAG (which activates PKC, which in turn phosphorylates calponin) [81, 114]; and sarcoplasmic/endoplasmic reticulum calcium ATPase (SERCA) transporters which fill the intracellular stores with Ca2+ [76]. Accordingly, at least four genes encoding different calmodulins were preferentially expressed in ACCs (*Cirobu.g00014804*, LogFC = 3.2; *Cirobu.g00000306*, LogFC = 2.8; *Cirobu.g00009220*, LogFC = 2.3; *Cirobu.g00004386*, LogFC = 1.5). Furthermore, ACCs also express *IP3 Receptor* (*Itpr, Cirobu.g00009359,* LogFC = 2.1), *Phospholipase C beta* (*Plcb, Cirobu.g00006467,* LogFC = 1.2), and *Atp2a* (alias *SERCA, Cirobu.g00010771*, LogFC = 2.9) genes. Notably, *Ciona* Atp2a shows highest similarity (~ 74%) with ATP2A2/SERCA2, which is the only SERCA pump expressed in mammalian smooth muscles [76]. However, many of these effectors are known to be expressed in neurons and sensory cells in various species, and the presence of secretory vesicles suggests the ACCs [121] could also have a neuromodulatory or endocrine secretory function in addition to contractility. However, their relative depletion of genes involved in synaptic vesicle dynamics, such as *Snap23/25* (*Cirobu.g00013764,* LogFC = − 1.3, Pct-1 = 0.25), and *Synapsin* (*Cirobu.g00007591,* LogFC = − 1.3, Pct-1 = 0.17), argues against a classic neuronal identify for the ACCs (Additional file 2).

**Fig. 3:**
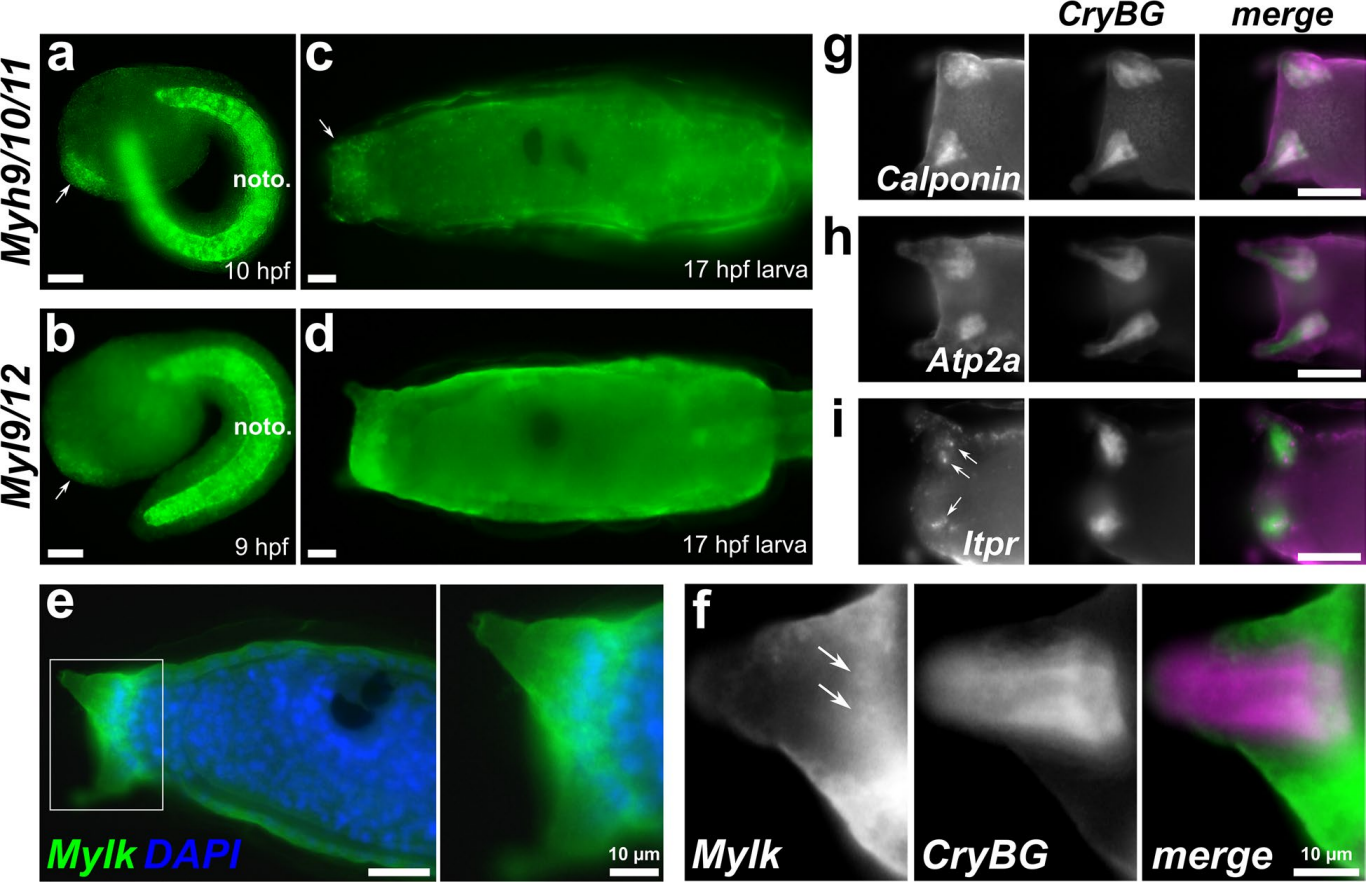
In situ hybridization for smooth muscle-like effector gene expression in ACCs. **a***Myh9/10/11* at 10 h post-fertilization (hpf), showing expression in papilla progenitors (arrow). Expression is also strong in the notochord (noto.). **b***Myl9/12* at 9 hpf, showing expression in papilla progenitors (arrow) and notochord. **c***Myh9/10/11* expression in 17 hpf larvae, showing faint but broad signal in the papilla territory (arrow). **d***Myl9/12* expression in 17 hpf larvae, showing almost undetectable expression above background in papillae, a bit more in notochord. **e***Mylk* expression (green) at 17 hpf, counterstained with DAPI to label DNA (blue). Inset of boxed area shown right, indicating broad expression in the papilla territory. **f** Two-color in situ hybridization with *Mylk* (green) and *CryBG* (magenta) probes show strong *Mylk* expression in cells surrounding the protuberances, but also more weakly in the ACCs, which are labeled specifically by *CryBG*. Sub-cellular mRNA localization of *Mylk* appears quite different from that of *CryBG*, but still appears to localize around nuclei of ACCs (arrows). Top right: Two-color hybridization of *CryBG* (green) and **g***Calponin,***h***Atp2a,* and **i***Itpr* (all magenta) showing ACC-specific expression. Nuclear dots (arrows) indicate likely active transcription of *Itpr.* All scale bars = 25 µm unless otherwise annotated

To get a sense of the ACC-specific expression of these genes, we re-clustered and re-analyzed our single-cell dataset (Fig. 2, Additional files 1: Fig. S1, 3). This revealed that some were more broadly expressed (e.g., the myosin heavy and light chain genes), while others such as *Calponin, Itpr,* and *Atp2a/SERCA* appear to be more specific to the ACCs. To confirm these scRNAseq data, we performed whole-mount mRNA in situ hybridization. Expression of *Myh9/10/11* and *Myl9/12* was seen broadly in the entire papilla territory starting at the mid-tailbud stage (Fig. 3a, b), though *Mylk* was not detected at this stage (not shown). At the larval stage, *Myh9/10/11* was still broadly though weakly expressed in the papillae (Fig. 3c), while *Myl9/12* expression was broad but almost undetectable above background levels (Fig. 3d). Expression of *Mylk* at the larval stage was also broadly expressed in the papillae (Fig. 3e), being especially pronounced in cells surrounding or in between the three papillae presumed to be collocytes [120, 121], but two-color double in situ hybridization with *CryBG* probe and scRNAseq visualization (Fig. 2) suggested expression in the ACCs as well (Fig. 3f).

In contrast to the broadly expressed markers above, we found that by in situ hybridization the ACCs strongly and specifically express *Calponin* (Fig. 3g) and *Atp2a* (Fig. 3h), the latter of which has been previously documented [20]. Expression of *Itpr* was just as specific albeit a bit more faint, in the form of nuclear dots that usually indicate active transcription (Fig. 3i), though the in situ hybridization is not quantitative and subject to sub-cellular localization of transcripts and therefore relative staining of different probes cannot be used a proxy for differences in actual expression levels. Specific expression in ACCs versus other papilla cell types was confirmed by two-color, double in situ hybridization along with *CryBG* probe. Of these three genes, only *Atp2a* is expressed outside the ACCs, specifically in tail muscles (Additional file 1: Fig. S2). Taken together, these data suggest that some smooth muscle effectors are highly and specifically expressed in the ACCs, although others (including the non-muscle/smooth muscle myosin chains and Mylk) are more broadly and/or precociously expressed in the entire developing papillae.

### The ACCs of *Ciona* papillae show contractile behavior and calcium transients during larval settlement

**Fig. 4:**
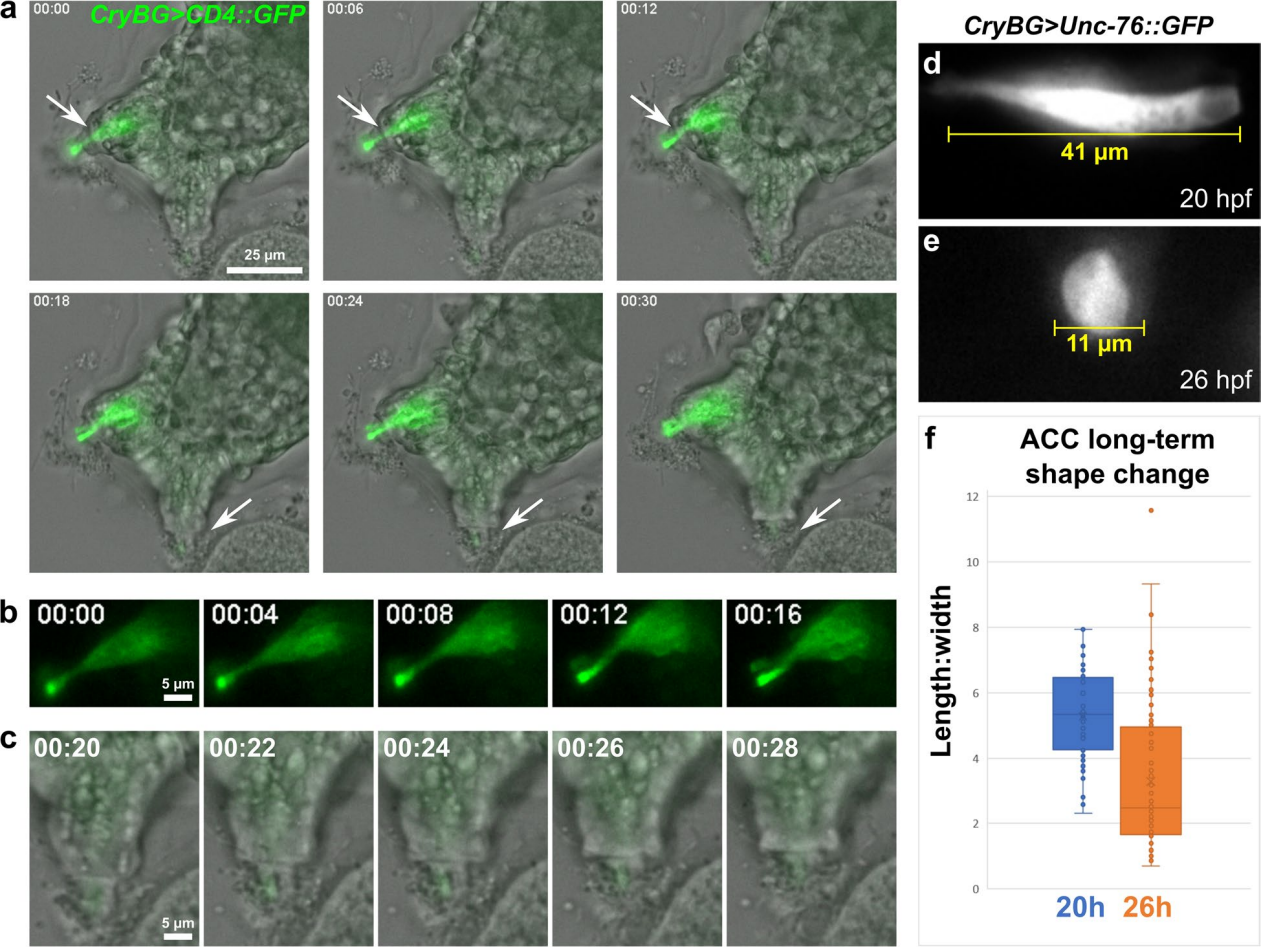
The contractility and shape change of ACCs during papilla retraction. **a** Still images captured from live time-lapse video (one animal recorded, see Additional file 4: Video S1) of attached larva ~ 21 h post-fertilization (hpf). ACCs marked by *CryBG *> *CD4::GFP* expression. Right dorsal papilla (top arrows) retracts from 6 to 12 min timepoints, while unlabeled left dorsal papilla (bottom arrows) retracts from 18 to 24 min. **b** Same GFP-labeled papilla in **a**, but only GFP channel shown, revealing ACC contraction. **c** Same unlabeled papilla in **a**, showing the finger-like apical protrusion of the ACC retracting into papilla. **f** Representative image of an ACC labeled by *CryBG *> *Unc*-*76::GFP* at 20 hpf, indicating typical length at this stage prior to settlement. **e** representative image of labeled ACC at 26 hpf, indicating extreme rounded shape observed in many larvae at this stage, after settlement and metamorphosis begins. **f** Quantification of length:width ratio of ACCs sampled from 20 vs. 26 hpf larvae (*n* = 54 for 20 hpf, *n* = 84 for 26 hpf)

The expression of these smooth muscle-like effectors hinted at the possible contractile nature of the ACCs. Contractility is a well-documented property of the complex, eversible papillae of tunicate species with large, adultative larvae [17]. In fact, similar axial cells in the papillae of *Distaplia occidentalis* were observed to contract after papillary eversion [17], and regularly arranged microfilaments also support a putative contractile function for axial cells in the papillae of both *Distaplia* and *Diplosoma* spp. [110]. However, the contractile cells of these large papillae have not been characterized at the molecular level. Furthermore, the contractility of *Ciona* papilla cell types has also not been investigated in detail. Using live, time-lapse fluorescence imaging we observed the ACCs of late larvae, around the time they should be settling and metamorphosing (Fig. 4a, Additional file 4: Video S1). In larvae either attached to glass coverslips or to debris in the dish, we observed a relatively slow (on the order of several minutes’ duration), but obvious contraction of the ACC cells within each papilla. The contraction appeared to draw the finger-like apical protrusions [121] of the ACCs inwards (Fig. 4b, c), with likely mechanical resistance from both the secreted material (putative adhesive substances) surrounding these protrusions and the cells immediately surrounding them. This was highly reminiscent of the contractile axial cells of *Distaplia,* which contract over the course of ~ 30 min during papilla retraction [17].

Although the contractions in the time-lapse videos could be observed over the span of several minutes, we followed the ACCs further through larval settlement, using carefully staged preparations of fixed larvae electroporated with the ACC reporter plasmid *CryBG *> *Unc*-*76::GFP* [13, 92]. The Unc-76 tag is frequently used to fill the entire cytoplasm volume while excluding labeling of the nucleus [99]. We quantified ACC shape changes over the course of settlement, showing that they irreversibly transition from an elongated shaped to a rounded one between 21 and 26 h post-fertilization (hpf, 20 °C, Fig. 4d–f). However, it is not clear how much is due to active contraction versus other possible mechanisms of cell shape change. This irreversible change is expected, given the transient nature of the tunicate larva. From the time-lapse videos, we propose that the early stages of papilla retraction are driven by active contraction, but that later stages of ACC rounding may involve other processes such as de-adhesion or cytoskeletal rearrangements.

**Fig. 5:**
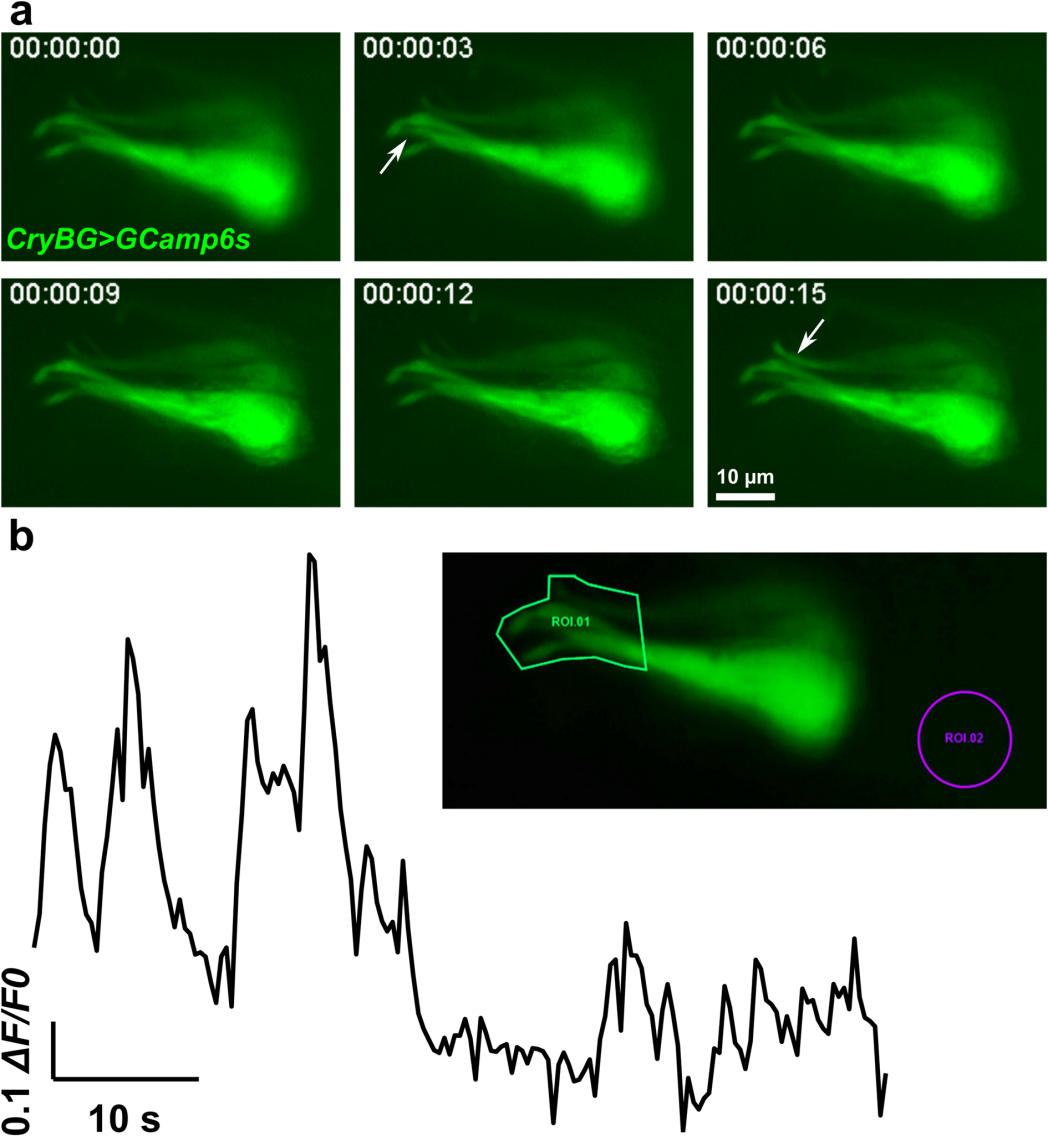
Intracellular calcium imaging in ACCs. **a** Still images captured from live time-lapse video (see Additional file 5: Video S2) of one larva electroporated with *CryBG *> *GCamp6s,* which drives expression of the calcium indicator protein GCamp6s. Transient increases in fluorescence intensity is seen in apical protrusions (arrows) around 3 s and 15 s in different cells. **b** Quantification of change in fluorescence in same time-lapse video, in the region of interest (ROI) 1 surrounding the apical protrusions of the ACCs (see inset), given as ΔF/F0 normalized to the maximal absolute value (1.0). See Additional file 6 for raw and normalized data

Calcium plays a central role in regulating smooth muscle contractions, primarily through the calmodulin–MLCK–myosin pathway, which promotes actomyosin contraction in response to intracellular calcium release. Such calcium transients can be seen during the stimulation of mammary gland myoepithelial cell contractions by the neuropeptide oxytocin [22]. We therefore used the genetically encoded calcium indicator GCamp6s [12] to visualize calcium dynamics in the ACCs. We observed and quantified several calcium transients in the ACCs, as indicated by the increase in GCamp6s fluorescence (Fig. 5, Additional files 5: Video S2, 6). It is unclear how the calcium dynamics we observed in ACCs in vivo relate to contractility, and they might reflect an upstream sensory function that might contribute to later ACC contractions via sustained calcium signaling.

### The ACCs express an orphan 7-transmembrane receptor that is enriched at their exposed apical tip

**Fig. 6:**
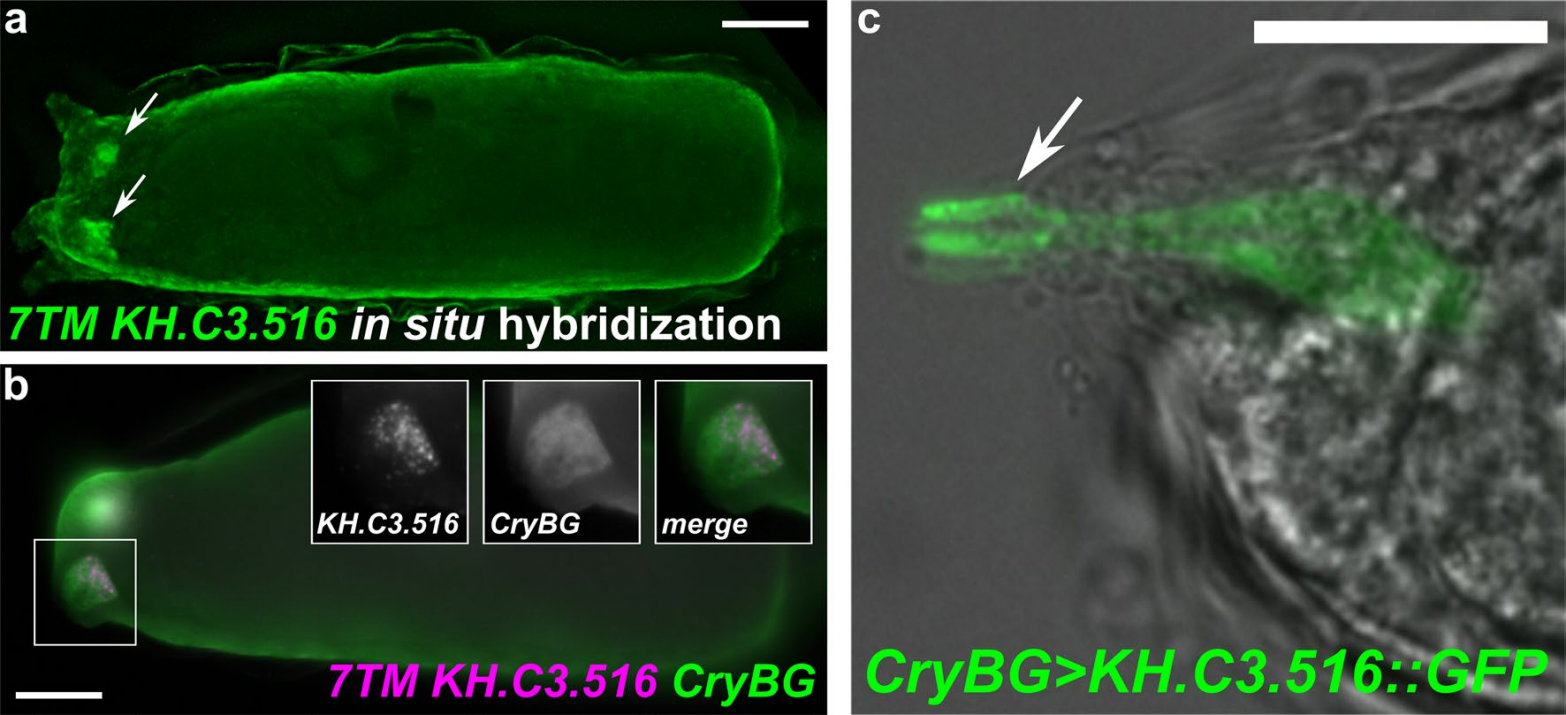
Expression of a putative orphan G protein-coupled receptor in the ACCs. **a** In situ hybridization showing expression of seven-transmembrane (7TM) protein-encoding gene *KH.C3.516* in the ACCs (arrows). **b** Two-color in situ hybridization with *KH.C3.516* (magenta) and *CryBG* (green) shows highly specific, strong expression in the ACCs (insets). **c** Expression of a KH.C3.516::GFP fusion driven by the *CryBG* promoter shows enriched localization in the apical tips of ACC protrusions (arrows), which are exposed to the environment through the tunic. All scale bars = 25 µm

What might be the cue to stimulate ACC contraction during settlement? In vertebrates, myoepithelial and smooth muscle cells are stimulated to contract via mechanisms that are distinct from typical skeletal muscle contractions. Instead of depending on stimulation by ionotropic neurotransmitter receptors and excitation–contraction coupling, mammary gland myoepithelial cells rely on binding of oxytocin to its receptor, a G-protein-coupled receptor (GPCR) that stimulates PLC activity to produce IP3, which in turn promotes calcium release via IP3 receptors to promote myosin activity. A similar mechanism is seen in smooth muscle cells [116], which also signal through DAG for maintenance of slower tonic contraction via Calponin phosphorylation by PKC [114]. The specific expression of *Plcb, Itpr,* and *Calponin* in the ACCs suggested they could be stimulated via a similar GPCR-dependent pathway. We found that the 2nd-most highly enriched (after *CryBG*) transcript model in our scRNAseq ACC transcriptome is the gene model *KH.C3.516* (*Cirobu.g00005701*). We found this model to be incompletely annotated, but searching upstream for the start of open reading frame we found this gene encodes a 7-transmembrane (7TM) pass protein (the typical structure of GPCRs) of unresolved phylogeny. It has no significant BLAST hits in other metazoans, but shows similarity to other predicted 7TM receptors in *Ciona*, at least two of which are arrayed in tandem immediately 3′ to *KH.C3.516*. Phylogenetic analysis available on ANISEED suggests it is related to a large number of proteins in various tunicate species, most of which encode 7TM proteins with no BLAST hits outside of tunicates (Additional file 1: Fig. S3) [58]. This suggests this gene may belong to a distinct family of 7TM-encoding genes that have expanded specifically in tunicates. ScRNASeq and in situ hybridization both revealed that *KH.C3.516* is highly and specifically expressed in the ACCs (Figs. 2, 6a, b).

The ACCs are surrounded by various cells including sensory neurons, which could be stimulating ACC contraction via neurotransmitter release. Therefore, one possibility is that *KH.C3.516* encodes a GPCR whose ligand could be a neurotransmitter released by neighboring sensory neurons. Alternatively, the ACCs might have external sensory capabilities themselves, hinted by the fact that their apical finger-like protrusions extend far through the adhesive “hyaline cap” and the tunic and are thus exposed to the outside world in a way that other papilla cells are not [35, 75, 121]. A fusion between KH.C3.516 protein and GFP (KH.C3.516::GFP) was found to be enriched at the apical tip of the ACCs (Fig. 6c), which would be consistent with a possible role for this GPCR in transducing unknown external cues via direct contact with potential substrates for settlement.

### Orthologs of vertebrate cardiomyocyte regulators are expressed in the papilla lineage

Shared functions or expression of “effector” genes between two different cell types (either in the same organism or in different species) are not necessarily indicative of descent from the same cell type in the ancestor, nor of deep homology due to evolutionary co-option of a gene network [4]. They can also indicate convergent evolution. A more robust support for homology (either classic or “deep”) can come from analyzing the expression patterns of the conserved, “core regulatory complex” (CoRC) that regulates those effector genes [4]. In the model recently proposed for the evolution of different muscle types, a CoRC composed of Gata, Fox, NK, SRF/Mef2, and myocardin family transcription factors regulated the expression of smooth muscle-like effectors in an ancestral smooth muscle type before the split between deuterostomes and protostomes [8]. Thus, we probed the expression of *Ciona* orthologs of these factors in the papilla lineage.

**Fig. 7:**
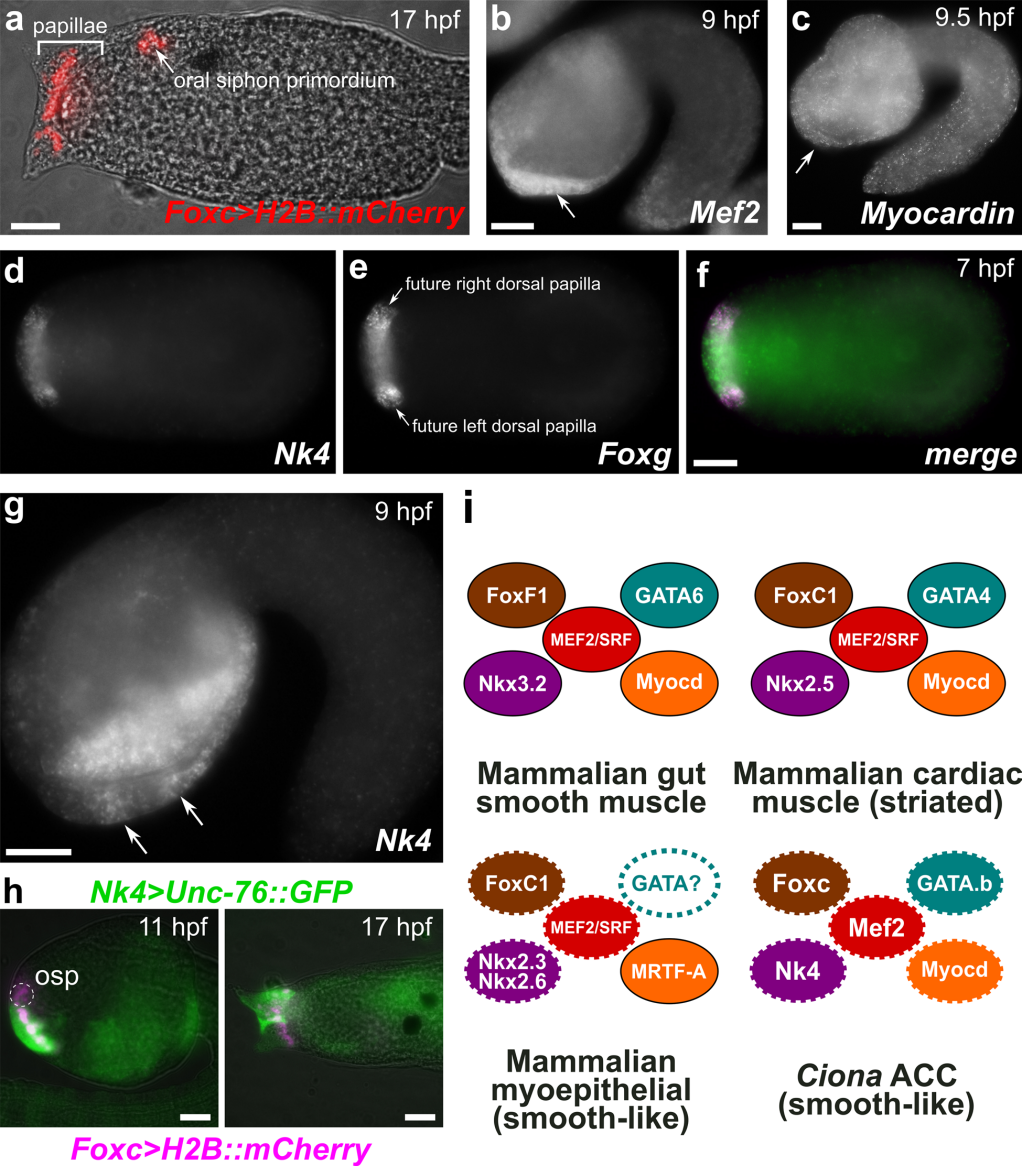
Cardiomyocyte/smooth muscle-like core regulatory complex (CoRC) factors in the ACCs. **a** Larva electroporated with *Foxc *> *H2B::mCherry* (Cirobu.REG.KhL57. 96,067-98,197, red), recapitulating expression of Foxc in the cell lineage giving rise to both the oral siphon primordium and the papillae. **b** In situ hybridization for *Mef2,* showing broad, possibly maternal expression throughout the embryo at 9 hpf, but also upregulation in the papilla territory (arrow). **c** In situ hybridization for *Myocardin,* which is expressed ubiquitously at 9.5 hpf, including in papilla territory (arrow). **d**–**f** Two-color in situ hybridization at 7 hpf for **d***Nk4* and **e***Foxg.* Presumptive protuberances of the papillae (which include the ACCs) are marked by *Foxg* expression at this stage, surrounding a territory of *Foxg*-negative cells. **f** Merged image shows *Nk4* (green) expressed broadly in both *Foxg *+(magenta) and *Foxg*-negative cells within the whole papilla territory. **g** In situ hybridization for *Nk4* at 9 hpf, showing gradual restriction to three presumptive papilla protuberances, two of which are visible at this focal plane (arrows). **h** Electroporation of *Nk4* (Cirobu.REG 4,056,723-4,057,773, green) and *Foxc* (magenta) reporter plasmids confirms expression of *Nk4* throughout papilla territory but not the oral siphon primordium (osp), viewed at 11 hpf and 17 hpf. Oral siphon primordium not visible in 17 hpf image. *Nk4* reporter mosaicism resulted in one unlabeled papilla. **i** Diagram comparing CoRCs for gut smooth muscles, cardiomyocytes, and myoepithelia to Ciona ACCs, suggesting deep homology according to the model proposed by Brunet et al. [8]. Dashed outline around solid color indicate expression data only, no functional data yet obtained. Dashed outline around white indicate no expression or functional data so far. All scale bars = 25 µm

The papillae of *Ciona* are derived from the a8.18 and a8.20 cell pairs of the medial anterior neural plate [112]. These cells are marked by expression of *Foxc* (*Cirobu.g00012813*), and give rise to the papillae and the oral primordium [112] (Fig. 7a). Interestingly, FoxC1 is proposed to have been part of the ancestral cardiac CoRC specifying smooth cardiomyocytes in the last common ancestor of protostomes/deuterostomes, which would have independently evolved striations in chordates and insects [8]. Although *Ciona* Foxc is the sole ortholog of human FOXC1 and FOXC2, it shows greater similarity to the former (~ 81%) than to the latter (~ 58%).

Because of this potential evolutionary connection, we investigated in more detail the expression of orthologs of other vertebrate cardiomyocyte CoRC genes. *Mef2* (*Cirobu.g00014543)* is the sole *Ciona* ortholog of vertebrate *Mef2* family genes. Although broad *Mef2* signal has been reported throughout the embryo and likely represents maternal expression [49], our in situ hybridization revealed stronger signal specifically in the papilla territory (Fig. 7b), suggesting zygotic upregulation in these cells. In contrast, the sole *Ciona Myocardin* ortholog *(Cirobu.g00006140*) had not been reported in *Ciona* before. According to RNAseq data on ANISEED, *Myocardin* expression appears to be constitutively expressed throughout embryogenesis, perhaps also maternally provided as mRNA. Indeed, by in situ hybridization, we detected faint but ubiquitous staining in most cells, including the papilla progenitors, at 9.5 hpf (Fig. 7c).

We also revisited the expression of *Nk4* (*Cirobu.g00009121*, the sole *Ciona* ortholog of vertebrate *Nk4* family genes including *Nkx2.5*), which has previously been observed in the papilla territory during development [48]. Using two-color, double in situ hybridization, we show that *Nk4* is expressed at 7 hpf in the presumptive papilla territory, in both Foxg+ cells that give rise to the protuberances as well as Emx+/Foxg-negative cells that do not (Fig. 7d–f) [61]. By 9 hpf, *Nk4* appears to become a bit more spatially restricted to each papilla protuberance primordium (Fig. 7g). These cells were followed through development by co-electroporating embryos with *Foxc* and *Nk4* reporter plasmids, which were co-expressed in the whole papilla territory as predicted by in situ hybridization (Fig. 7h). In 17 hpf larvae, we observed that *Nk4* reporter labeled the cells surrounding and in between the protuberances that also express *Mylk* at the larval stage (Fig. 3e, f).

We did not revisit the expression of *GATA4* orthologs in *Ciona,* the tunicate-specific paralogs Gata.a (*Cirobu.g00012060*) and Gata.b (*Cirobu.g00014947*). Gata.a shows roughly equal similarity to human GATA4, GATA5, and GATA6 (~ 56–58%), while Gata.b shows highest similarity (~ 83%) to GATA4, according to ANISEED. Gata.a is maternally loaded and is required for specification of the entire ectoderm [83]. Gata.b (*Cirobu.g00014947*) is also maternally expressed, but is upregulated zygotically at the late gastrula stage in the anterior epidermis, which includes the papilla progenitors [49, 71]. However, its function in papilla development is unknown.

Taken together, these expression patterns suggest that all the conserved cardiac CoRC factors are expressed at one point or another in the lineage that gives rise to the ACCs (summarized in Fig. 7i). Whether or not they are all co-expressed as proteins and bind as a single complex upstream of smooth muscle-like effector genes in the ACCs, or if they cooperatively regulate effector gene expression in an asynchronous manner (i.e., some acting as pioneer factors), remains to be tested. It should be noted that none of these were enriched in the ACCs at the transcript level according to our scRNAseq data, which was collected at 20 hpf, well after ACC differentiation.

## Discussion

Here we have described the contractile nature of the ACCs of *Ciona*, which appear to drive the retraction of the papillae by decreasing their length over several minutes. We have not observed these contractions to occur repeatedly—they occur only once and appear irreversible. This behavior, coupled to their distinctive shape and position in the center of each papilla, suggests homology to the ACCs of *Distaplia,* which undergo a similar, irreversible length-wise contraction over ~ 30 min to drive papilla retraction after eversion [17]. This slow, sustained contraction is similar to the “tonic” contractions of various vertebrate smooth muscles, as opposed to rapid, rhythmic “phasic” contractions [30, 122]. In vertebrates, this tonic contraction is partially dependent on lower activities of both MLCK and myosin phosphatase [37, 40]. Interestingly, we did not detect myosin phosphatase gene expression in the ACCs, which would be consistent with a tonic smooth muscle phenotype. The papillae of *Distaplia* are eversible, and this additional behavior appears to be driven by a distinct myoepithelial cell population surrounding each large cup-like papillae [17]. In contrast, the smaller papillae of *Ciona* are non-eversible. We conclude that, while *Distaplia* might contain an additional papillary myoepithelial cell type for eversion that is lacking in *Ciona,* both species have tonically contracting ACCs that drive the irreversible retraction of the papillae following larval attachment. The molecular profile of *Distaplia* ACCs and surrounding myoepithelial cells has yet to be investigated, but it will be interesting to see if they share the same suite of smooth muscle-like effectors that the *Ciona* ACCs express.

The presence of apical finger-like protrusions that extend out of the tunic has previously led others to focus on primarily a sensory role for the ACCs in *Ciona* or other species [74, 94]. Although our gene expression profiling suggests that the ACCs are contractile myoepithelial cells, we believe they still carry out some sensory function. This is consistent with the ACC-specific expression and subcellular localization of *KH.C3.516* that is part of a large tunicate-specific family of 7TM proteins. Perhaps these distinctive 7TM proteins encode a set of tunicate-specific chemosensory receptors, analogous to vertebrate olfactory receptors. One hypothesis is that the ACCs use chemical sensing to transduce environmental signals that would directly stimulate ACC contraction and palp retraction upon contact with a suitable substrate. This possibility is suggested by the presence of Ca2+ waves and the expression of effectors like PLC and the IP3 receptor, which might be acting downstream of GPCRs like that encoded by *KH.C3.516.* Papilla retraction in turn might aid permanent attachment by bringing the larva and its secreted adhesives in closer contact with the substrate. Another possibility is that the contractility of the ACCs could be used for a mechanosensory function, for instance to probe substrate rigidity, which has been shown to influence tunicate larval settlement [31]. In this case, the ACCs might generate force by pulling on the substrate (attached by the adhesive hyaline cap secreted by the collocytes), while adjacent papilla neurons might sense the mechanical resistance of the substrate. These are not mutually exclusive functions: ACCs could use their chemosensory ability to pull on candidate substrates, which in turn would stimulate adjacent mechanosensitive neurons to operate as an AND logic gate. Finally, it is also possible that the ACCs do not partake in any sensory functions, either directly or indirectly, and that their sole function is to drive papilla retraction in response to stimulation by adjacent sensory neurons.

The finding that the ACCs express orthologs of vertebrate non-muscle/smooth muscle contractility genes suggests they may be a homolog of vertebrate smooth muscle-like cells, particularly myoepithelial cells (see below). Although some of these effectors are more broadly expressed in the papillae (*Myh9/10/11, Myl9/12,* and *Mylk*), or their transcription start in the progenitors of the entire papilla territory at the tailbud stage (*Myh9/10/11* and *Myl9/12),* only the ACCs express these in addition to other classic myoepithelial markers, especially *Calponin/Transgelin.* It will be important to ascertain whether the more broadly expressed effectors carry out additional functions in the other cell types of the papilla, like morphogenesis or adhesive secretion. This is a distinct possibility, given that these myosins are equally related to both smooth- and non-muscle myosins in vertebrates. Alternatively, seemingly precocious “primed” transcription of *Myh9/10/11* and *Myl9/12* might be necessary for proper ACC development, similar to other cell lineages in *Ciona* [79].

Vertebrate myoepithelial cells are smooth muscle-like cells that are specified by smooth muscle regulatory factors like myocardin [59] and express smooth muscle contractility genes but retain their organization in an epithelium [63]. They are also usually derived from non-neural surface ectoderm but not neural crest. These are all consistent with the development and morphogenesis of the ACCs, which are derived from surface ectoderm anterior to the neural tube, and remain arranged in an epithelium, forming tight junctions with other cell types in the papillae such as the collocytes [121]. It is unclear if the ACCs are homologous to any particular type of myoepithelial cell in extant vertebrates. The papillae of tunicate larvae secrete adhesive substances [120] and might share a common origin with various secretory glands in vertebrates. The most obvious candidate would be the cement gland of various vertebrates, though myoepithelia have not been reported in these glands. However, other dermal glands have myoepithelial cells that use their contractility to help release secreted material, e.g., mammary, lacrimal, or salivary glands. A recent scRNAseq analysis of mouse salivary glands revealed that many smooth muscle effector genes or their close paralogs are enriched in the myoepithelial cells when compared to other salivary gland cell types. *Transgelin, Myl6, Myl9, Mylk, Myh11, Myl12a, Myh9, Myl12b, Desmoplakin,* and *Plcb4* were found to be among the top 500 most highly enriched transcripts in myoepithelial cells [93]. This analysis also revealed myoepithelial cell-specific enrichment of transcripts encoding conserved regulators of smooth muscle/cardiomyocyte fate, like *Foxc1, SRF, and Mef2a.* Myoepithelial cells are also found in the eye, specifically the muscles of the iris. The iris dilator muscle is a smooth muscle-type myoepithelium, while the iris sphincter muscle (pupillary constrictor) loses its epithelial arrangement later in development [62]. Unlike the tunicate ACCs, mammalian iris muscles are thought to be derived from neurectoderm, while in chick they derive from neural crest [21, 29, 56, 69, 104]. In humans, mutations in FoxC1 cause Axenfeld–Rieger syndrome, which is characterized by a wide range of congenital malformations of the iris and cornea [109]. Thus, it may be interesting to reassess the embryological origins of iris muscles and the roles of FoxC factors in their development.

We found that orthologs of all the vertebrate cardiac CoRC factors (FoxC/F, NK4, SRF/MEF2, GATA4/5/6, and Myocardin) are expressed in various intermediate progenitors that will eventually give rise to the ACCs. Although we note that it is not clear whether all these factors are present as proteins in the ACCs and whether or not they bind DNA as a single complex, it is not clear they do so in vertebrates either. For instance, FoxC1 and FoxC2 are expressed early in vertebrate cardiac development and are required for downstream expression of other CoRC genes like *Nkx2.5* and *Mef2c* [57, 119], suggesting some of these factors might not function as a literal protein complex but more like a conserved subset of a gene regulatory network. Additionally, GATA mRNA expression is downregulated in Nkx2.5+ cardiomyocyte progenitors [53], also indicating that CoRC gene expression in vertebrates may be just as dynamic as we see in the ACC lineage. Given the rapid development of *Ciona* larvae (less than 24 h) and our lack of antibodies to detect CoRC proteins, it is difficult to compare such temporal dynamics between *Ciona* and vertebrates. Even if some of these factors are preciously expressed in *Ciona* relative to vertebrates, it may be due to the peculiarities of the fast-developing *Ciona* embryo and the transient nature of larval cells like the ACCs. For instance, several *Ciona* larval tail muscle effector genes initially require direct binding by Tbx6 factors, which are first expressed in paraxial mesoderm progenitors but not in committed myocytes [118].

In the proposed model of secondary striation of vertebrate cardiomyocytes, an ancestral cardiac CoRC was proposed to be composed of FoxC, NK4, SRF/MEF2, GATA4/5/6, and Myocardin, and that this regulated *smooth* muscle effectors, not striated muscle effectors [8]. Curiously, this evolutionary connection may be reflected in the transient expression of smooth muscle effectors in immature cardiomyocytes in vertebrates [84, 117]. It is important to note that cardiomyocytes in tunicates are also striated, not smooth [73]. If the conserved CoRC factors expressed in the papilla lineage do indeed regulate the expression of smooth muscle effectors there, it would lend support to the idea that the ACCs share a common origin with smooth muscles and cardiomyocytes, but prior to the latter becoming secondarily striated in the last common olfactorian (tunicates + vertebrates) ancestor. A close evolutionary relationship between ACCs and vertebrate vascular smooth muscles is another possibility. Effector gene expression in vertebrate vascular smooth muscles is regulated by a CoRC similar to that of cardiomyocytes, with the possible exception of NK3 factors replaced for NK4 [10, 60, 115]. Although there are no reports of vascular smooth muscles in tunicates, the ectoderm-derived extracorporeal vasculature of certain species carries out peristalsis via epithelial contractility [18, 23]. Cephalochordates lack a central heart with striated cardiomyocytes, and instead rely on vessels surrounded by a single layer of myoepithelial cells to drive circulation. Thus, early chordates may have co-opted an ancestral myoepithelial cell type to elaborate their vascular system.

It should be noted that Foxf, but not Foxc, is expressed in the cardiopharyngeal lineage that gives rise to cardiomyocytes in *Ciona* [5], as well as in the heart/kidney complex of hemichordates [33], and the dorsal–ventral muscles of planaria that co-express Nk4 and GATA4/5/6 [90]. Similarly, Foxc has not been implicated (to our knowledge) in cardiomyocyte development outside of vertebrates. Therefore, it may be that the role of FoxC in cardiomyocyte development represents a vertebrate innovation. In contrast, Foxf factors are found in gut smooth muscles in vertebrates, planaria, and *Platynereis* [8, 90], and in the striated gut muscles of *Drosophila,* which are proposed to be secondarily striated [8]. Interestingly, in *Drosophila* the *FoxC* ortholog *crocodile* is expressed in midgut muscle founder cells, which fuse with myoblasts derived from cells expressing the *FoxF* ortholog *biniou* to form the longitudinal midgut muscles [52, 65]. *Foxc* and *Foxf* genes are found in an ancient cluster that is conserved throughout bilateria [66]. Thus, one possibility is that these genes might have had overlapping functions in ancestral smooth muscle cell types, which later become subfunctionalized.

## Conclusion

Given all these considerations, the ACCs of *Ciona* and various glandular myoepithelial cells in vertebrates might be derived from an ancestral smooth myoepithelial cell type. In turn, such an ancestral myoepithelial cell could share an even more ancient common origin with various smooth muscle cell types, as well as with cardiomyocytes. Under this scenario, the evolution of different smooth-type contractile cells would predate the expansion and subfunctionalization of smooth muscle effector genes in vertebrates. To further support this hypothesis, it will be important to ascertain whether additional smooth muscle/cardiomyocyte CoRC components also regulate smooth muscle effectors in vertebrate myoepithelial cells. The *Myocardin*-related transcription factor MRTF-A was shown to be required for mammary gland myoepithelial cell differentiation and effector gene expression [59]. Other CoRC factors such as Nkx2.3 and Nkx2.6 (both of which are orthologs of *Ciona* Nk4, like their paralog Nkx2.5), Mef2a, SRF, and FoxC1 are expressed in developing salivary glands and salivary myoepithelial cells in mammals [7, 93], but the roles of such smooth/cardiac CoRC orthologs have not been determined in these cells.

Just how deep is this myoepithelia–cardiomyocyte evolutionary connection? One intriguing possibility is that it goes back to the bilaterian ancestor. In the nematode *C. elegans,* the pharyngeal muscles are not striated and are also epithelial in nature [64]. Furthermore, the evolutionary connection between the nematode pharynx and the heart (which nematodes do not have) has been postulated before based on the requirement of the NK4 homolog CEH-22 for pharyngeal muscle development in *C. elegans* [72]. Alternatively, the ACCs (and perhaps the extracorporeal vasculature of some species) might represent a much more recent, tunicate-specific co-option of an ancestral cardiovascular smooth muscle program in surface ectoderm cells.

Of course, it could also be that the expression of the above factors in the Ciona papilla lineage does not reflect evolutionary conservation of an ancestral myogenic gene regulatory network. ACC specification and differentiation is promoted by transcription factors like Islet and Foxg [61, 113], which are not part of any myogenic CoRC. Since the contractility machinery assembled in the ACCs could result from various evolutionary co-options and convergences, this stresses the need for comprehensive studies comparing gene regulatory networks in more species and myogenic cell types. Incidentally, our study indicates that understanding the specification and differentiation of myoepithelial cells might be crucial for better models of muscle type evolution. As a direct descendent of an ancestral myocyte or as a unique cell type evolved de novo in tunicates, there is much to still learn about the ACCs and their uncommon combination of contractility, epidermal surface location and potential sensory role.

## Supplementary information

**Additional file 1.** Figures S1–S3 and protein/DNA sequences.

**Additional file 2.** Differential gene expression analysis table for Cluster J (ACCs) from Sharma et al. [91].

**Additional file 3.** Differential gene expression analysis table, after reanalysis of Sharma et al. [91].

**Additional file 4.** Timelapse video of papilla contracting (Video S1).

**Additional file 5.** Timelapse video of Gcamp6s fluorescence in ACCs (Video S2).

**Additional file 6.** Raw and normalized Gcamp6s imaging data.

## Data Availability

The datasets and methods supporting the conclusions of this article are available in the Open Science Framework (OSF) repository, https://osf.io/5dc4u/.
