# Supplementary material for: Expression of smooth muscle-like effectors and core cardiomyocyte regulators in the contractile papillae of Ciona
Source: EvoDevo. 2020 Aug 3;11:15. doi: 10.1186/s13227-020-00162-x (PMC7397655; doi:10.1186/s13227-020-00162-x)
Supplement: Supplementary file 1 — Additional file 1. Figures S1–S3 and protein/DNA sequences. [file 13227_2020_162_MOESM1_ESM.docx]

Additional File 1 – Johnson et al.

**
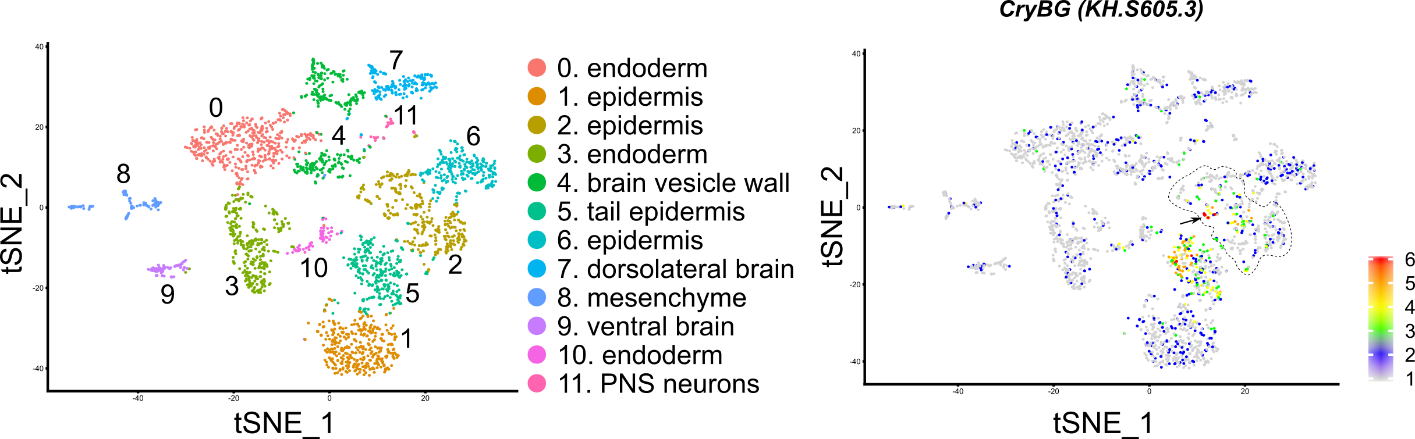
**

**Fig. S1**

Left: re-clustering of scRNAseq data from Sharma et al. (2019). Right: differential expression of *Beta/gamma Crystallin (CryBG)* superimposed on clustering diagram, with putative ACCs indicated by black arrow. Cluster 2 (dotted outline), representing different cells embedded in mostly the head epidermis, was selected for re-clustering data shown in Figure 2. *CryBG* is also expressed in tail epidermis cells (Cluster 5). See Additional File 3 for differential gene expression data.

**
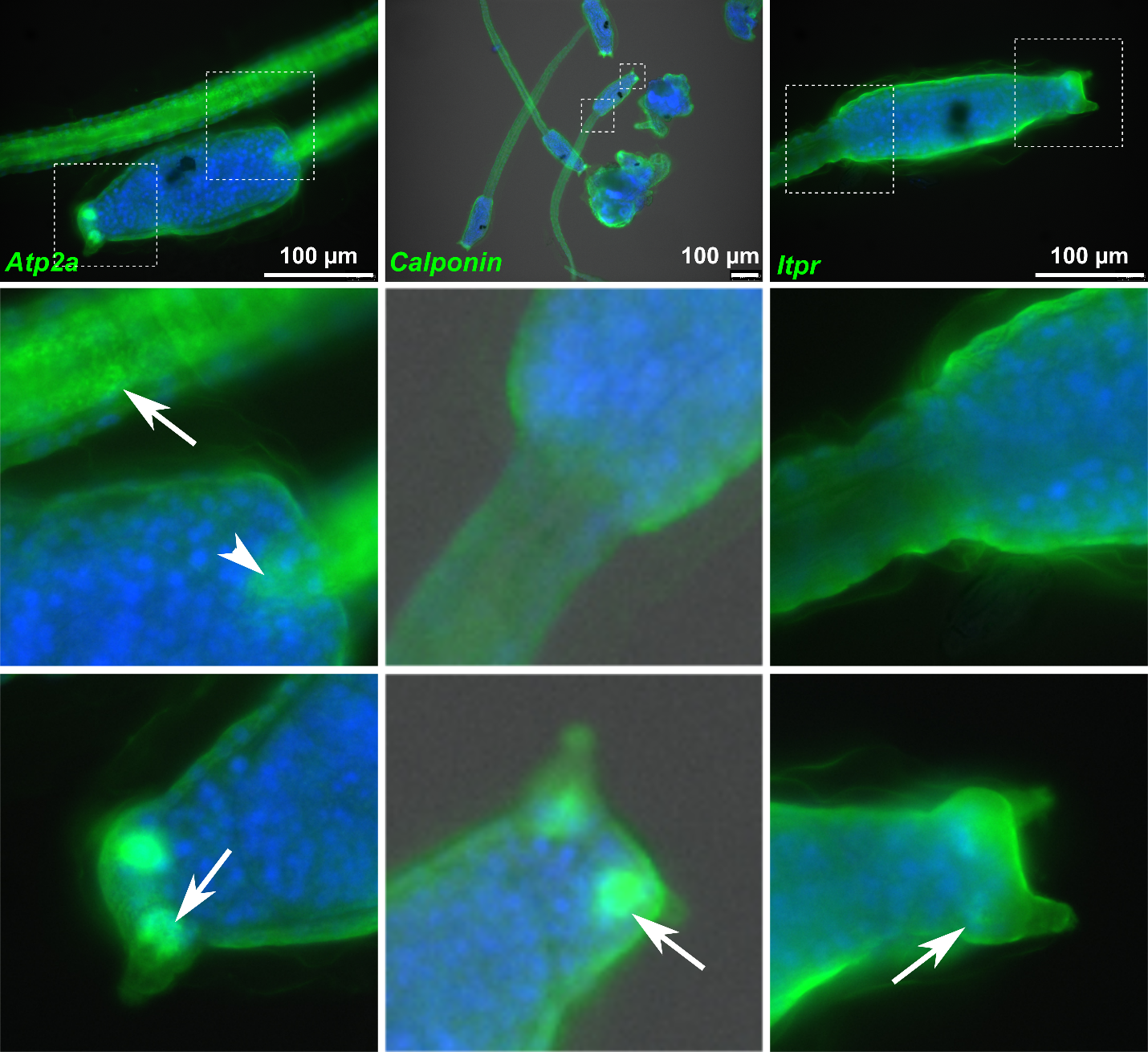
**

**Fig. S2**

Whole-larva views of *in situ* hybridizations for a) *Atp2a,* b) *Calponin/Transgelin,* and c) *Itpr.* d) *Atp2a* shows expression outside the ACCs, in the tail muscles (arrow), seen most clearly in at the base of the tail muscles where autofluorescence from the tail tunic is not present (arrowhead). Equivalent region in larvae stained for e) *Calponin* and f) *Itrp* does not show tail muscle expression. g-i) Magnified views of signal in ACCs (arrows) for all three genes. *Itpr* shows faint but specific signal (see **Figure 3i**). d-i inset regions indicated by dashed boxes in a-c. Nuclei counterstained by DAPI.


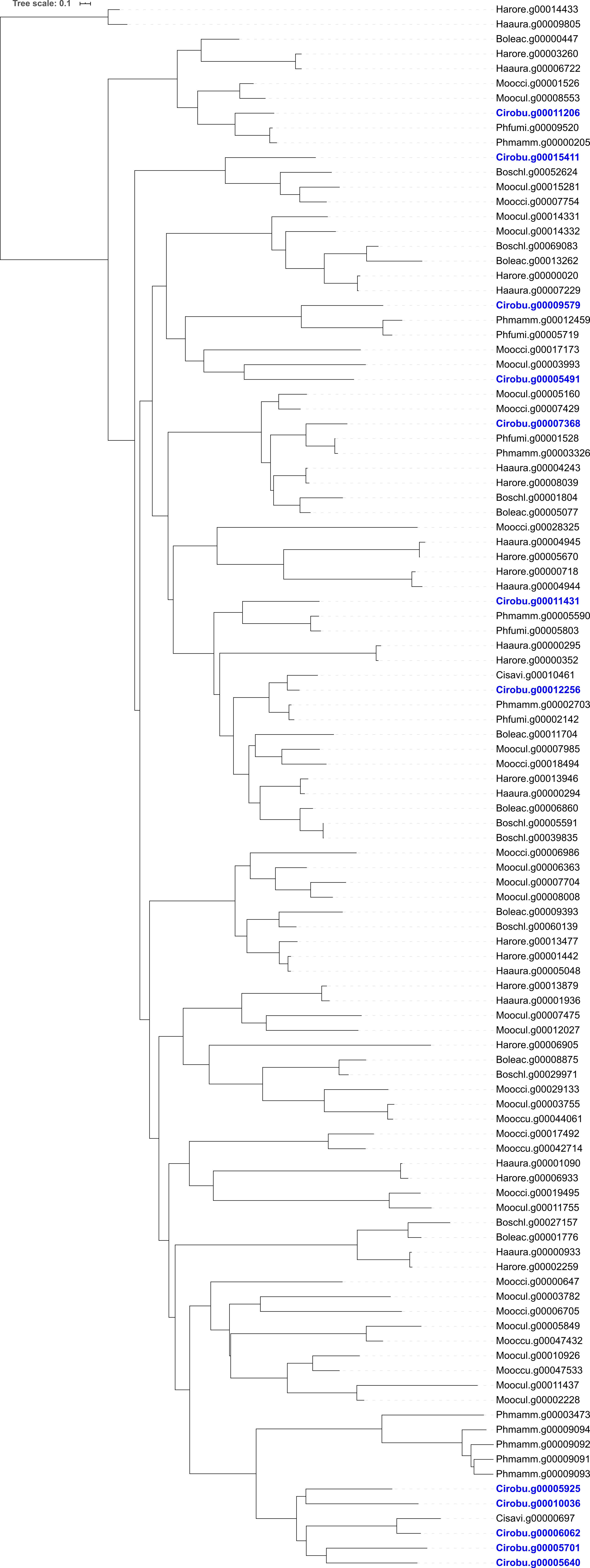


**Fig. S3:** Phylogenetic tree drawn using Interactive Tree of Life (iTOL) application, based on phylogeny provided by ANISEED. In blue font are those sequences from *Ciona robusta* (Cirobu). Boleac: *Botrylloides leachii*, Boschl: *Botryllus schlosseri*, Cisavi: *Ciona savignyi***,** Haaura: *Halocynthia aurantium*, Harore: *Halocynthia roretzi*, Moocci: *Molgula occidentalis*, Mooccu: *Molgula occulta,* Moocul: *Molgula oculata*, Phfumi: *Phallusia fumigata*, Phmamm: *Phallusia mammillata*

**7TM-KH.C3.516 and closely related sequences from C. robusta. Red sequences indicate those missing from the predicted KH gene model but included based on manual inspection.**

>Cirobu.g00005701-KH.C3.516

MEISGNITTMTNFLLTSDVSSATELNVAQGKVLRASEDMMVVALIVLSLYLTVASFAYQWKQPSLSALKGTNRLCSACAVMLLVQSLFFEVEMRVSSPTLQLCYAYYVIVLILATINKSLTYCVLWVRQRSFYKNPKSQCMVYFGNAVLTGIIVLPILQVGVLMLNIVDTSPVGCITRKGPEFSTVLVPFVFMLFVVVQLILLGLVLRPIVRHIMRNKIGQTSNLTTVAVRLSVCTTLCVFTDLAFLTVAIIQPKNVGFSFGPIVFHFNSTVNVAAMLCSFADYSERLMPFSPSNNSSVLEEDGQEPNTEKSRV

>Cirobu.g00005925-KH.C3.722

MSNGSSNDSAKTTMSDDNLDFYHHPVTAHVALGFLILCSTYLLVATVYYQKRHAKRKCCSNALFIAASVILLIESAWFETEVLQTSSTSLFCTAYYAINATSATLIRTLVYGVLWLRQKELYRNTVTHLHNKCVAIMGKIILAGIVIFGLFQVVVLLVTPQTGTEHGCVTSKPTPVVKILTPVVFTTYSLFQFLLLGLTLYPVVQHMCGKAEQRNQRVLEAIVVRLSVCTFVCVLCDISFMIATIKRNQSMPYNFIPIAFAFNSLINLFVSLCSFVDYEWRLCPFRPPRVTSTTVSSRSGSEHFRRTHTAVSNTDYTKATAKEDKNHSKQGIPI

>Cirobu.g00005640-KH.C3.458

MSTATATFNISTTAVMNITVAGNPYMIAEECIVALLLAFSLYGLIVAAVFAVRCKVAALRKENILFVLSVGILFLRACFFEVEIHVPASTTAFCVAFSAINVVLANINRGIVYLILWMRQRTFYQSPILTHLNSKYIKVLSKTILIGIVTMPIILIGMLVAIPIQFNLDLSCHTKRSNIFLQIAVPVVYALQALIQIFLLAIVLYPIIKHIIITKFKRNNKMASVIIRLSVSTTVCVLTDIAFTAFSINPSENYSFYFLAIIYGINTLINVCAVAFSYTDYLHRILPCIKKKQQPVSPEASAVRETNLAKTIP

>Cirobu.g00006062-KH.C3.851

MNQSTRQYNTTQLSLSVQPQASTLQEGILSRLPIVTTTPDSSNVTNNGTVIQPRHVPLFDTIAVHTLIVISCYLLIATIIYTVRKKKDKVAPHLHRVNALCVVATSLLLFESCWFLVEVYVTGSATFCTAYSATNVTVGTINRAVIYTILWLRQRAFYRKRPTACATSRTINLLSNALLGGILMFALIQIMTMSSIPILSVKGKCVTGKPSGFLNIFAPIIFFIIALFQVVLLLPILYPVVKHVHSRRTGQNKGRLDSVITRLCISGSVCVLTDIGFLILSANDHPSVSFSFIPICYAFNTVINVTSTIMSFSDFSNRLLPLRCGCVHKKEASLIPKEIAHQLTVSHI

>Cirobu.g00010036-KH.C9.486

MFPLSLRNHLRIYCTTFPQRISMHVEVSCIACQVNQFLSMNISGLSDSFEWASYTYASEHRMYPPLLFSFLPRITFSKYRKVLTLHESTMSSQQILLSEQTTNYAEKSFTEMPVVKNISVQFNLHLLNSRIAEQIIITVLIISSLYLSLACLAYSSKYFNLNPKLKHSNLAICFVSCLLFPQCIWFEAEVFLTPKTEMFCVAYTVINATIGTITRTIIYMVLWWRQYALYQHQLEYLLSRFIRMVSRSTFVGIFTFAAVQIVSLSLVPQEVIDSSCRSCEIPAILGVILPMVFTISSLFQVVLLALTLYPIILHMLRDKTTPSNIRMDSVVLRLACCTGACVFCDFVFVLSIRLKPQTSSYNLIPVLYGYNSLFNSIAALCSFADYKTRLFPFRYEKAYQPSFNGNNIPTQAEASITPYELEYSVEI

>Cirobu.g00011206-KH.L141.50

MRHHFFPTTTTYTNTEVTTDEIISTNISGLRIPFTIIGLLFSVASLYVFLSITIHRLRAIKRQKKEEIAQRQQSVRSRRSKGTSPYMDNLSVVASMFAFMNCVSDQPLLYIKSPTNVTCKIIQSLSFVLYSAGIVCLYSFLWFRQRACYKNPTLSEMHGKFARILSSTILPLFIIMMLAGMILTGALWEFTTSDIGCIEVAQTRTPLIVLFTGAVAMQGTLLTLFVYPLKRHAGFATSNVLPSDKLLMALVKRSIITTGLCVLADALAFIIAVALPTNTPFIVTNLLYDANLVCNVV

CVVGSFADWRQRLAPWLRNNANRCELIGSMSRSSRTTALTTTTVV

>Cirobu.g00015411- NCBI:KH.101242726

MSQNKSAANVTYREFSSLQWIACELTSCIFSVACCYVLICLLVYEIRMRTQSVDQGVATVSGDNSIVQQSVRSDRRKAKKTRRNQKWLRYFTILCTLCAVLKVLVNQIDLFWGTKSSIACTLFSITSICGYGGSLFFIYIILWLRQYIVQSHSALRHLPHRNIRVYGGRILLGVMLTLFGSLFVLNFVTIKVRHSPDGCIFADSGEVSQSARSLAGLIFTIVFQPAFAVLLLYPLLKKLPSHRRPKLTSTSIRPAHPGAKKCNSVEQVKSLIRRTLIAIALVTVSDIGAIVATFLLEKQPIIFVALAYDANAFVGIVSAIYSFTDWRQRFLGKKTPNADVEKTV

>Cirobu.g00009579-KH.C8.90

LYTESSTLQVPGNNTEFFKLRPRGAQWILYAIIVLLFAVAVYLFVVVVYYVVFRPLKVGQKNESKTQKGYGDGTASKSRSIYKIPMLVLCFFMAVLCVVFFTVQQFIIFAKTRPATLILVIKFVFGTKIVLGYAFMWLRQRLFYTGPLRRLHRKNSPLLILSIVSLVFILVNIVAQIVLTWYWSAISARLAEMISWLVGCTLVQSTLLCLFVYPLWKQNSEKRRSLRSKSGNEALSNLTSGIDKLIRRSLIIACIWFTSDILDILVQFASEVINGYNTVFLHNLVLNDLNAIVNTVCLVASFNNWKSIMFPWWKSTKPTEENTVKETVTSQIPVTHA

>Cirobu.g00005491-KH.C3.32

MLIVVVIQSFLGLYICVVLVYYECQAMPKNLGRIQRKAKRLRCLLTFCATVGVVYFSIALTAIINSRFLQSNQACQVLVVAQKLTYSVVLYSLYGFLWLRQKMFYDNPSMENLYNRKLRIASWGVFVLAILCFLIQAVLTLLGSKYQVKNKPPGVCYEQERWLSELETLSVLHRSNADPAAVPSLTPRTLRKEFQNSFKRHASIGATSNRGRKNERRLPLLIRRCVILTSICVMVNIAATVLTATLYKSENKLATVLYGANLLVNLSCSVGFFRQWRQIIFPPSVRCRNPEEDVSMDATRNSIDSRSEYAKIRISRKITNSSDVRV

>Cirobu.g00007368-KH.C5.48

MNATKFEITPTTTVLSKLNHTEYQEWNLLGQKVWTSESKATWSPNKDVAWYTCEAIHITCIVVAFYILYALVHFRILRKAEVAKTSARLRKGGRWLENLCIMGVLTALLRFGNNQALLFTVDSTSRCSTLLNVSIALYNVTIHPIYTFLWVRLRIFYSNRALKHLYSKTVRFLSWVALLCFICVTMVCMVLLIVDRDNSASGRVCANTNSNDSSTVPANSSRPPHQQGVRVNIMTGLLGGCIQLSFLALFLYPIMDNKLRAVRANHTRGKSVALITLIRRSFVLAGLCILSDVAIALLIKVVYTSHPSAIFAPLAMYDVNLVVNLLCIVMTFRRWRVMLFPWFYTSRCPFAACPSACHHRLQPGSSRTYIVNKKTPVAPLASVESRSQTWDRSTSHFSSRRRREKKLQIPEIREPVPDVGLPVLREISEEKEAEWKSQSLSLQNHKIESELQQYSDTVFQLSYPEFERQRSTSLVLRTENKCSDKKSKTALAASLIAKSKCWMRDNKQNNT

>Cirobu.g0001143-KH.L152.52

MVSDHTVAFVGWRNNTTCIRTVNVSTALYAPSIYSIYVFLWFRQNEFYSNPILKHLANKHLIRFSRVTILLLFIAGFAVTVLFQIPEVTGWDFKATPSGCIDVADIADIEALPTILVVITIVGQVALLGLLVYPILVTKKNQQQMTSSRSVTSPKVDRSRPLLQQAIVGRMRERGSVGMDAARKSSDVIDDVGSSDDDISHSELCWSEGESTDCESQAGDITMHGSTRGHRFTRRDGGSPAPRRVASLGSPKRIKVRKLRRTPRKSSMRKREQKLTILIKKVSILASICVASDVTATILQIMISLPELVYFFIFDVNLLINTVCVLLSFKQWRKIFFPRFTPPTPQPHDPPSSGGNTTNTHLPNHDPHGRNLDNVSIRSGLSTHTEMIVMSRSGTPEVHDVTRV

>Cirobu.g00012256-KH.L25.4

MKILGNLSDLKNLTDIDGKPKEPFSELYWIFSQIVLSIVIIFSLYLLVCLSRYATITKCSPGREISGKKGKMLYRLCLVSLVMAVARFVSDEAVAITGWTNDKNCVTTVSVSAVFYSLSLYPIYIFLWMRQSIFYANPVLSHILNPVVTFISWATLVVMLSGGAVLTVIYILPEITGWRYEATDSGCRDAADVSGFDLVTCLVVAFTVSFQVSLLALFMYPLLTKKTQRYRSQSTRAARANTNAENASASYEENSVPEKNSDEVSSSEPKRVVSDEDHTKVAVSYVGKPPHRGGYFNTKSVFYRKTQKKDGGDKTKKGIFRLSHVSTTSFTDLMKKPKSPSNASTSEGAKKSKRGKTQQGRRIIQFMRKAFFLTLFCVMSDVIFAVIQMVVIVPELVYLVLFDVNLLINIVCIVMSFRDWPRMIAPVCARHLIQDKNQSRDSNTNGRSTRTMVSRLAAANTRLTPQKRPNLSSSSEATANALP

**Sequences of unpublished plasmids and probes used in this study:**

CryBG -1068 to -24 (Cirobu.REG.KhS605. 16789-17833) KH.C3.516 coding sequence GFP

>CryBG>KH.C3.516::GFP (hypothetical, not fully sequenced)

ggcgcgcctaattcttactgttcggttgaaactcttgaatccactatgacgtcatcatcgtcgctacaaccgctataaacagcgagaaacaaaataaaaacaatcgacttcattacggaagagttgggtcgaaagtggttgttgcatgaaatatatgtgtgttatatctcggtgctaagtggtacaacaaacaaataaaacgtctggtttatgagatgctcgcgtggtacgggctatgtgatgtcataatacctttgtctattatccttgttacgacataattaccatgaaacagcattgtgacgtcacagtgctgtttgtgttggacaaacaacacgtggtttctacgagggggaatcccctctctgtcattattacgtatgatgacgtataatatcgattattattcaccatccaaggttatatacgatatctatttggttttatccgcttggtggcgacatcaatcctcaactacaattggtattatgacgtagttcgtagttacgtcacaagtcacacaacaaagaatttattatgagcaaataaacgaactaatatgacgcaacaataactaacaatttatcacgtgatcgttttatttattttgtttgttatgtcataaccacttggtgcttaaaagggagcttatgacgtattaatgaactttaattattaaaacaatcttgcttatcgtgctgtgttacgtcataatacatgcattagcaattactttgttttgtcataatatgaattatgtaattatcgtgacgttgtttgttttaaaaacccatttattattacgtcataatactttagatggttgtgtatgttacgtcataatgttaattgttgaataaaaatgaagaaacaaagccatgaagaaggcggtttgttataaaacattgttgaagggatggaattaattatgttgttaacgtttgaattattgatgtaacaaggacgacaagtgagcgcaaatcagatttcgtttactttccttctaaccctcctaaccactgcttatttcgcattttgtacaatcgaagtttcgcggccgcaaccatggaaatctccggcaacattacaacaatgacaaattttctgcttaccagtgacgtgtcgagtgcgactgaacttaatgtggctcaaggaaaagttctaagagcgtccgaggacatgatggtggttgcactaatcgtgctcagtttatacttaacagtagcctcctttgcttaccaatggaagcagccgtcgctcagtgccttgaaagggacaaacagattatgcagcgcctgtgcagttatgcttctggtccagtcgttgtttttcgaagttgaaatgcgtgtcagttctccgaccttacagctctgttatgcgtactacgtaatcgtgctaattcttgccacaatcaacaagtcactaacatactgtgtactttgggtacgacaaaggagcttttacaagaatcctaaaagtcagtgtatggtgtacttcggcaacgcagtacttacaggaatcatcgtgctgccaatcttacaggttggggttttaatgcttaacattgttgacacgtctcccgtagggtgcataacaagaaaagggccggagttctcgacagttttagtcccgttcgtatttatgctcttcgtagttgttcaactgattctgcttggcctcgtcctccgcccaattgtccgccatatcatgcggaataagatcgggcaaacttccaacttgacgacggttgcagttcgtctcagcgtttgcacaacactctgcgtttttaccgaccttgcatttttgacagtagcgataattcaaccaaagaacgtgggatttagttttggaccaatcgtctttcactttaactcaactgtcaatgtggcagctatgctgtgttcgtttgctgattactctgaacgtcttatgccgttttcaccatccaacaactccagtgttttagaagaggatggccaggaaccaaacacagagaaatctcgagtcactagtaccatggtgagcaagggcgaggagctgttcaccggggtggtgcccatcctggtcgagctggacggcgacgtaaacggccacaagttcagcgtgtccggcgagggcgagggcgatgccacctacggcaagctgaccctgaagttcatctgcaccaccggcaagctgcccgtgccctggcccaccctcgtgaccaccctgacctacggcgtgcagtgcttcagccgctaccccgaccacatgaagcagcacgacttcttcaagtccgccatgcccgaaggctacgtccaggagcgcaccatcttcttcaaggacgacggcaactacaagacccgcgccgaggtgaagttcgagggcgacaccctggtgaaccgcatcgagctgaagggcatcgacttcaaggaggacggcaacatcctggggcacaagctggagtacaactacaacagccacaacgtctatatcatggccgacaagcagaagaacggcatcaaggtgaacttcaagatccgccacaacatcgaggacggcagcgtgcagctcgccgaccactaccagcagaacacccccatcggcgacggccccgtgctgctgcccgacaaccactacctgagcacccagtccgccctgagcaaagaccccaacgagaagcgcgatcacatggtcctgctggagttcgtgaccgccgccgggatcactctcggcatggacgagctgtacaagtaagaattccagctgagcgccggtcgctaccattaccagttggtctggtgtcaaaaataataataaccgggcaggccatgtctgcccgtatttcgcgtaaggaaatccattatgtactatttaaaaaacacaaacttttggatgttcggtttattctttttcttttacttttttatcatgggagcctacttcccgtttttcccgatttggctacatgacatcaaccatatcagcaaaagtgatacgggtattatttttgccgctatttctctgttctcgctattattccaaccgctgtttggtctgctttctgacaaactcggaacttgtttattgcagcttataatggttacaaataaagcaatagcatcacaaatttcacaaataaagcatttttttcactgcattctagttgtggtttgtccaaactcatcaatgtatcttatcatgtctggatcgacaaagtcaaagcggccatcagatctgccggtctccctatagtgagtcgtattaatttcgataagccaggttaacctgcattaatgaatcggccaacgcgcggggagaggcggtttgcgtattgggcgctcttccgcttcctcgctcactgactcgctgcgctcggtcgttcggctgcggcgagcggtatcagctcactcaaaggcggtaatacggttatccacagaatcaggggataacgcaggaaagaacatgtgagcaaaaggccagcaaaaggccaggaaccgtaaaaaggccgcgttgctggcgtttttccataggctccgcccccctgacgagcatcacaaaaatcgacgctcaagtcagaggtggcgaaacccgacaggactataaagataccaggcgtttccccctggaagctccctcgtgcgctctcctgttccgaccctgccgcttaccggatacctgtccgcctttctcccttcgggaagcgtggcgctttctcaatgctcacgctgtaggtatctcagttcggtgtaggtcgttcgctccaagctgggctgtgtgcacgaaccccccgttcagcccgaccgctgcgccttatccggtaactatcgtcttgagtccaacccggtaagacacgacttatcgccactggcagcagccactggtaacaggattagcagagcgaggtatgtaggcggtgctacagagttcttgaagtggtggcctaactacggctacactagaaggacagtatttggtatctgcgctctgctgaagccagttaccttcggaaaaagagttggtagctcttgatccggcaaacaaaccaccgctggtagcggtggtttttttgtttgcaagcagcagattacgcgcagaaaaaaaggatctcaagaagatcctttgatcttttctacggggtctgacgctcagtggaacgaaaactcacgttaagggattttggtcatgagattatcaaaaaggatcttcacctagatccttttaaattaaaaatgaagttttaaatcaatctaaagtatatatgagtaaacttggtctgacagttaccaatgcttaatcagtgaggcacctatctcagcgatctgtctatttcgttcatccatagttgcctgactccccgtcgtgtagataactacgatacgggagggcttaccatctggccccagtgctgcaatgataccgcgagacccacgctcaccggctccagatttatcagcaataaaccagccagccggaagggccgagcgcagaagtggtcctgcaactttatccgcctccatccagtctattaattgttgccgggaagctagagtaagtagttcgccagttaatagtttgcgcaacgttgttgccattgctacaggcatcgtggtgtcacgctcgtcgtttggtatggcttcattcagctccggttcccaacgatcaaggcgagttacatgatcccccatgttgtgcaaaaaagcggttagctccttcggtcctccgatcgttgtcagaagtaagttggccgcagtgttatcactcatggttatggcagcactgcataattctcttactgtcatgccatccgtaagatgcttttctgtgactggtgagtactcaaccaagtcattctgagaatagtgtatgcggcgaccgagttgctcttgcccggcgtcaatacgggataataccgcgccacatagcagaactttaaaagtgctcatcattggaaaacgttcttcggggcgaaaactctcaaggatcttaccgctgttgagatccagttcgatgtaacccactcgtgcacccaactgatcttcagcatcttttactttcaccagcgtttctgggtgagcaaaaacaggaaggcaaaatgccgcaaaaaagggaataagggcgacacggaaatgttgaatactcatactcttcctttttcaatattattgaagcatttatcagggttattgtctcatgagcggatacatatttgaatgtatttagaaaaataaacaaataggggttccgcgcacatttccccgaaaagtgccacctgacgtctaagaaaccattattatcatgacattaacctataaaaataggcgtatcacgaggcccttacgtattaattaa

Nk4 intron 1, partial (Cirobu.REG.KhC8.4056723-4057773) basal promoter of FOG (Cirobu.REG.KhC10.1827572-1827787) Unc-76::GFP

>Nk4>Unc-76::GFP

ggcgcgccctgcccggcgcctacaacgtcaacatcaagttggacatcacctcccacaacgaggactacaccatcgtggaacagtacgaacgcgccgagggccgccactccaccggcggcatggacgagctgtacaagcacgggtaagttgttttacttgtctcgttgctttaaaagcagcttacgcatgaataagcggaatgggaattagctgaataagatctacgagcctttatgaccctgactatatttagtaaataaaactcctggaccagcgcgttatattttgaaggcaggtagacgtgtcaagtttcactagcgtgagtcggtttaacaaaatatacttaatgcgaagcggagcgaatcgcgggaccaggaaacaaatgccgtgaaccgtgcagggtacaattaacaagcgatacttgacatgtggtttggcatagtttagcggcatattagcaccaaaggggcacccaaacaaatcacacgagcgactttccatgcagttatgggcgacagtgacagacaaagccacaactcggtggcgccggcgttacgacttagaagcaggtcggccaatcacaacaaaaagccacagcgatggagatcggtgctaatgaaaacatagatgcgagtaatgcgttacaataacacggggcgaggaatcaggcagtcggcgactatcggaaacggctccagtgaatcgaagccccattgtcccagagcgcgacaagcgcctcgacaaacaagagaacaagagataattacaaattaacaagggtggcgatgtatggcgggccgtgtcgccgaattcaaatagcgcccacttgaaatgatcgcggtaattttggatagactctgtcgccgccattttgattatcttgttaagatttcaagtgtcggttgcatggtaagcttgttgaccaaaacaatatcgttagcggattacttgcacattggtcttgtttcgataaacaaacaactaaatcgatgcaaaatttagtgtcagtgcagcgcacacaaaaaaacgaatccgtctttccgcgaagctgtaaatgttgaaattcctttccataaaccgtaactggtcatattacgagaagtacatagagagttcaactcgctcaaaaactgtaaacacttgcgagtaaccagcaattataaccaaccgcaaaacgaagtgtctcgagcagctgaagcttgcatgcctgcaggtcgactctagaggatccggcaaagcttcgtgtattgtaccggcccattgtcaatcatgcaaacttgatattatattgacaagagaagaaggcagtttaaattaaaactctaaagtagagagacattaatctcagctgacaaggcaggtggtcacagtaagttcatttaaatagttggccaacaatagcctttccaagaaagtatttttgttccaggtctatacaaaaataacacacaacatggcggccgcaaccatggcggatctgcgagtaccggacattccgctcgcctcgtgtgatgatgatgatatcgatagtaataagaatttgagcaaccattcatcagacgagaaacatcactgcaacagcaacagcgacgaggaacgtcttcatgacgagttctctggatcccttgaggaccttgtcggcaactttgacgaaaaaattgcggcatgcctgaaggaccacgaggtgacgacagcggatattgcacctgtgcagatacgtactcaagaggaagttatgaatgaaagccaaacatggtggacattaaccggaaactttggaaacattcaacctctcgactttggaacctcttcgatatgtaaaaagatggccgcagctctggacagtgattcattgaaagacgacgcatctacacgccgaagtatgacaaattccgatgatgaggatcttttacgacaacaaatggatgttcatcaaatgattggacatcatcatggatctacggatactggtggtgaaacacctccacagactgctgatcaagttatcgaagaaattgatgaaatgttacaggtaccggtcgccaccatggtgagcaagggcgaggagctgttcaccggggtggtgcccatcctggtcgagctggacggcgacgtaaacggccacaagttcagcgtgtccggcgagggcgagggcgatgccacctacggcaagctgaccctgaagttcatctgcaccaccggcaagctgcccgtgccctggcccaccctcgtgaccaccctgacctacggcgtgcagtgcttcagccgctaccccgaccacatgaagcagcacgacttcttcaagtccgccatgcccgaaggctacgtccaggagcgcaccatcttcttcaaggacgacggcaactacaagacccgcgccgaggtgaagttcgagggcgacaccctggtgaaccgcatcgagctgaagggcatcgacttcaaggaggacggcaacatcctggggcacaagctggagtacaactacaacagccacaacgtctatatcatggccgacaagcagaagaacggcatcaaggtgaacttcaagatccgccacaacatcgaggacggcagcgtgcagctcgccgaccactaccagcagaacacccccatcggcgacggccccgtgctgctgcccgacaaccactacctgagcacccagtccgccctgagcaaagaccccaacgagaagcgcgatcacatggtcctgctggagttcgtgaccgccgccgggatcactctcggcatggacgagctgtacaagtaagaattccagctgagcgccggtcgctaccattaccagttggtctggtgtcaaaaataataataaccgggcaggccatgtctgcccgtatttcgcgtaaggaaatccattatgtactatttaaaaaacacaaacttttggatgttcggtttattctttttcttttacttttttatcatgggagcctacttcccgtttttcccgatttggctacatgacatcaaccatatcagcaaaagtgatacgggtattatttttgccgctatttctctgttctcgctattattccaaccgctgtttggtctgctttctgacaaactcggaacttgtttattgcagcttataatggttacaaataaagcaatagcatcacaaatttcacaaataaagcatttttttcactgcattctagttgtggtttgtccaaactcatcaatgtatcttatcatgtctggatcgacaaagtcaaagcggccatcagatctgccggtctccctatagtgagtcgtattaatttcgataagccaggttaacctgcattaatgaatcggccaacgcgcggggagaggcggtttgcgtattgggcgctcttccgcttcctcgctcactgactcgctgcgctcggtcgttcggctgcggcgagcggtatcagctcactcaaaggcggtaatacggttatccacagaatcaggggataacgcaggaaagaacatgtgagcaaaaggccagcaaaaggccaggaaccgtaaaaaggccgcgttgctggcgtttttccataggctccgcccccctgacgagcatcacaaaaatcgacgctcaagtcagaggtggcgaaacccgacaggactataaagataccaggcgtttccccctggaagctccctcgtgcgctctcctgttccgaccctgccgcttaccggatacctgtccgcctttctcccttcgggaagcgtggcgctttctcaatgctcacgctgtaggtatctcagttcggtgtaggtcgttcgctccaagctgggctgtgtgcacgaaccccccgttcagcccgaccgctgcgccttatccggtaactatcgtcttgagtccaacccggtaagacacgacttatcgccactggcagcagccactggtaacaggattagcagagcgaggtatgtaggcggtgctacagagttcttgaagtggtggcctaactacggctacactagaaggacagtatttggtatctgcgctctgctgaagccagttaccttcggaaaaagagttggtagctcttgatccggcaaacaaaccaccgctggtagcggtggtttttttgtttgcaagcagcagattacgcgcagaaaaaaaggatctcaagaagatcctttgatcttttctacggggtctgacgctcagtggaacgaaaactcacgttaagggattttggtcatgagattatcaaaaaggatcttcacctagatccttttaaattaaaaatgaagttttaaatcaatctaaagtatatatgagtaaacttggtctgacagttaccaatgcttaatcagtgaggcacctatctcagcgatctgtctatttcgttcatccatagttgcctgactccccgtcgtgtagataactacgatacgggagggcttaccatctggccccagtgctgcaatgataccgcgagacccacgctcaccggctccagatttatcagcaataaaccagccagccggaagggccgagcgcagaagtggtcctgcaactttatccgcctccatccagtctattaattgttgccgggaagctagagtaagtagttcgccagttaatagtttgcgcaacgttgttgccattgctacaggcatcgtggtgtcacgctcgtcgtttggtatggcttcattcagctccggttcccaacgatcaaggcgagttacatgatcccccatgttgtgcaaaaaagcggttagctccttcggtcctccgatcgttgtcagaagtaagttggccgcagtgttatcactcatggttatggcagcactgcataattctcttactgtcatgccatccgtaagatgcttttctgtgactggtgagtactcaaccaagtcattctgagaatagtgtatgcggcgaccgagttgctcttgcccggcgtcaatacgggataataccgcgccacatagcagaactttaaaagtgctcatcattggaaaacgttcttcggggcgaaaactctcaaggatcttaccgctgttgagatccagttcgatgtaacccactcgtgcacccaactgatcttcagcatcttttactttcaccagcgtttctgggtgagcaaaaacaggaaggcaaaatgccgcaaaaaagggaataagggcgacacggaaatgttgaatactcatactcttcctttttcaatattattgaagcatttatcagggttattgtctcatgagcggatacatatttgaatgtatttagaaaaataaacaaataggggttccgcgcacatttccccgaaaagtgccacctgacgtctaagaaaccattattatcatgacattaacctataaaaataggcgtatcacgaggcccttacgtattaattaa

Foxc -2132 to -1 (Cirobu.REG.KhL57. 96067-98197) H2B::mCherry

>Foxc>H2B::mCherry

ggcgcgccccgcctacgtagggtaaataccgctcagggcgcgtacgttgcacacagcgggaaatgacaaagaaatggataaaaagatgcatggttttctaaattgtccggcatacagaaaacattcccctgcaaagttatatacgtggtaactcgtaagtgggaatgcggtgttataaaacaaaacacccatgttataacgaccgtcgttttctcggcacttgataataaataaatcgcattcattcaattaataatagatagccagcagcacgcgtatgttatttgagcaatcgcttgcaagaggcacgtccgggaagtccacaccttctacctgaaacgtctactttgacgttatgaacccgtgcaccgaaaagtgttagcgaaaccaagatggttaaactggaatagcttcctcaacacccacgtacactctttccttttgcgtcaagcggggtttcctttccctctagaagtgttgatataaatccttcatgacaccggacaccgcattaagcgcggttcaatcagaatatctatgcggcacccgcttcatgtagtaggccgggggaagatgggacacctttagcacataatacccaaacaacctaatcgtattttaaacagttaacaacggtctatggaagtcgtgaggatacggtttaataattctttaaatatttcttgtttactaccaaatgagacgagaaaatagaataaaaaagtgtcccatctccccccaccctactgtataaaccagaaaaagtggaaatgtccgaaaagagttttatcagcattttagtattttggcgatttaagctttagatacacaaggtgttagtaattgcggaaaggtgttttgttcagttaatctgacgaaaggagttggtttatatttttatttctaccaatatatatatattgctaccaggatttagtaaaaagcgttttatagttaatttaaaagtaaatattttaaacagtaagcaacaaatctgtacgattaattgctccgtaaacgtttactgcatgttcggttaaataaccacctcgcttttcatttttcaatcctactcgttaacgtcgttttgctaattccctttacttttttcaaggccaagttagttactataccaaacgcaatataacataacttaaactgttgtttagctatttaaatacagaaacaaaaataagattaattgaaatagcaaaccaagagaatcagcaacaaaactacacttgttaaaaatacgctatgaaggtaaaaaaaaactaagtaaaaatgtccaatatatttaataaaacgaagctatggtgggtggggaaaccttaagctaaatgctccaggaaaatatgaatcatcgacgcctaagttgccgccttagttgcataactcattgtatagcgagtcacggaaaatgctgcgcgtgtaaatttccgcatggtgtcgctgctgagccaaccggtctcgctcgtttcaaaaagtcgagttttaccgcaaaaaactcttcggttacatctcttatttataaacagcaacccgaggagtcacgctgtaaactgatcgggtcgtgacaaggttcggacaggagaggcagcttcagttataaccgctgaatatcaacggtgaacgttaaccgccatttttaatgaacgttggagttaaaaagttccaagattgagagattaatttaaaagttgtggtttatataaacagggctattgggtaaggctccatagtgagcggtgtcagcaggtgtttcgtaaggcggcgcgtgccaagttctctacttagagcttgtcaaaacacgatctaattactgcatcattagcgcgccattgttcctcgcgaaagttgattgggattatgacgctcctgctttccattgtttaaggggaagatgaactttttaccttcgctcaggctcgactcggtcgtgggcaggtaccggcagaaaacattcgattattgacacgaaggcagtgcgagtgttgtgagggaagtcgtttcggagcgacgtttgtttgcttgcagcgttggcgttcagattctaacttttatatatctcgggcagtgttagtgtaagttaagttacgttgaacacaggacaccgaatccttggtttgattctctatagcggccgcaaccatgccaccaaagcctgccagcaagggagctaagaaggccgccagcaaggcgaaagctgctcgcagcacggacaagaagcacaagagaaggcgaaaggaaagctactttatatacatatacaaagtgctgaagcaggttcacccggacacgggcatcagcggcaaagccatgtcaataatgaactcgttcgtcaatgacatctttgaacgaatcgcagccgaagcttctcgcctcgcccactacaacaagagatccacaatcaccagcagagaaatccagacggccgtcaggcttctgttacccggcgagttggccaagcacgccgtcagcgaaggcaccaaggccgtcaccaagtacaccagctcaaaggtcgacaggccaatctggccgcgggtcgacggtaccgcgggcccgggatccatcgccaccatggtgagcaagggcgaggaggataacatggccatcatcaaggagttcatgcgcttcaaggtgcacatggagggctccgtgaacggccacgagttcgagatcgagggcgagggcgagggccgcccctacgagggcacccagaccgccaagctgaaggtgaccaagggtggccccctgcccttcgcctgggacatcctgtcccctcagttcatgtacggctccaaggcctacgtgaagcaccccgccgacatccccgactacttgaagctgtccttccccgagggcttcaagtgggagcgcgtgatgaacttcgaggacggcggcgtggtgaccgtgacccaggactcctccctgcaggacggcgagttcatctacaaggtgaagctgcgcggcaccaacttcccctccgacggccccgtaatgcagaagaagaccatgggctgggaggcctcctccgagcggatgtaccccgaggacggcgccctgaagggcgagatcaagcagaggctgaagctgaaggacggcggccactacgacgctgaggtcaagaccacctacaaggccaagaagcccgtgcagctgcccggcgcctacaacgtcaacatcaagttggacatcacctcccacaacgaggactacaccatcgtggaacagtacgaacgcgccgagggccgccactccaccggcggcatggacgagctgtacaagtaagaattccagctgagcgccggtcgctaccattaccagttggtctggtgtcaaaaataataataaccgggcaggccatgtctgcccgtatttcgcgtaaggaaatccattatgtactatttaaaaaacacaaacttttggatgttcggtttattctttttcttttacttttttatcatgggagcctacttcccgtttttcccgatttggctacatgacatcaaccatatcagcaaaagtgatacgggtattatttttgccgctatttctctgttctcgctattattccaaccgctgtttggtctgctttctgacaaactcggaacttgtttattgcagcttataatggttacaaataaagcaatagcatcacaaatttcacaaataaagcatttttttcactgcattctagttgtggtttgtccaaactcatcaatgtatcttatcatgtctggatcgacaaagtcaaagcggccatcagatctgccggtctccctatagtgagtcgtattaatttcgataagccaggttaacctgcattaatgaatcggccaacgcgcggggagaggcggtttgcgtattgggcgctcttccgcttcctcgctcactgactcgctgcgctcggtcgttcggctgcggcgagcggtatcagctcactcaaaggcggtaatacggttatccacagaatcaggggataacgcaggaaagaacatgtgagcaaaaggccagcaaaaggccaggaaccgtaaaaaggccgcgttgctggcgtttttccataggctccgcccccctgacgagcatcacaaaaatcgacgctcaagtcagaggtggcgaaacccgacaggactataaagataccaggcgtttccccctggaagctccctcgtgcgctctcctgttccgaccctgccgcttaccggatacctgtccgcctttctcccttcgggaagcgtggcgctttctcaatgctcacgctgtaggtatctcagttcggtgtaggtcgttcgctccaagctgggctgtgtgcacgaaccccccgttcagcccgaccgctgcgccttatccggtaactatcgtcttgagtccaacccggtaagacacgacttatcgccactggcagcagccactggtaacaggattagcagagcgaggtatgtaggcggtgctacagagttcttgaagtggtggcctaactacggctacactagaaggacagtatttggtatctgcgctctgctgaagccagttaccttcggaaaaagagttggtagctcttgatccggcaaacaaaccaccgctggtagcggtggtttttttgtttgcaagcagcagattacgcgcagaaaaaaaggatctcaagaagatcctttgatcttttctacggggtctgacgctcagtggaacgaaaactcacgttaagggattttggtcatgagattatcaaaaaggatcttcacctagatccttttaaattaaaaatgaagttttaaatcaatctaaagtatatatgagtaaacttggtctgacagttaccaatgcttaatcagtgaggcacctatctcagcgatctgtctatttcgttcatccatagttgcctgactccccgtcgtgtagataactacgatacgggagggcttaccatctggccccagtgctgcaatgataccgcgagacccacgctcaccggctccagatttatcagcaataaaccagccagccggaagggccgagcgcagaagtggtcctgcaactttatccgcctccatccagtctattaattgttgccgggaagctagagtaagtagttcgccagttaatagtttgcgcaacgttgttgccattgctacaggcatcgtggtgtcacgctcgtcgtttggtatggcttcattcagctccggttcccaacgatcaaggcgagttacatgatcccccatgttgtgcaaaaaagcggttagctccttcggtcctccgatcgttgtcagaagtaagttggccgcagtgttatcactcatggttatggcagcactgcataattctcttactgtcatgccatccgtaagatgcttttctgtgactggtgagtactcaaccaagtcattctgagaatagtgtatgcggcgaccgagttgctcttgcccggcgtcaatacgggataataccgcgccacatagcagaactttaaaagtgctcatcattggaaaacgttcttcggggcgaaaactctcaaggatcttaccgctgttgagatccagttcgatgtaacccactcgtgcacccaactgatcttcagcatcttttactttcaccagcgtttctgggtgagcaaaaacaggaaggcaaaatgccgcaaaaaagggaataagggcgacacggaaatgttgaatactcatactcttcctttttcaatattattgaagcatttatcagggttattgtctcatgagcggatacatatttgaatgtatttagaaaaataaacaaataggggttccgcgcacatttccccgaaaagtgccacctgacgtctaagaaaccattattatcatgacattaacctataaaaataggcgtatcacgaggcccttacgtattaattaa

>CryBG in situ probe

TATTGATGTAACAAGGACGACAAGTGAGCGCAAATCAGATTTCGTTTACTTTCCTTCTAACCCTCCTAACCACTGCTTATTTCGCATTTTGTACAATCGAAGTTTCGTGGGTCGTTGCTGGTGGAACAATGGGCAAGATAATTTTATTCGAAGACGTTGAATTCGGGGGAAAAAAGTTGGAATTGGAAACCTCAGTGTCGGACTTGAACGTCCACGGTTTCAATGATATTGTGTCGTCGATTATTGTCGAAAGTGGAACTTGGTTCGTTTTCGACGACGAAGGGTTCAGCGGTCCGTCATATAAGCTTACCCCGGGGAAGTACCCTAACCCGGGATCATGGGGCGGCAACGACGACGAATTGTCATCGGTGAAACAACAATGATTTTTACGTCACAATAAATGTGAAAATAAAGAAGCTTTTGTTGCGTCATAA

>Myh9/10/11 in situ probe

GAATTGGAACAACAGCTTTTACAAGCTAACCCAATTCTTGAGGCTTTCGGAAATGCCAAGACCGTTAAAAACGACAATTCCTCCAGATTTGGAAAATTCATCAGGATTAATTTCGATACATCTGGTTACATTGCTGGAGCTAATATTGAGACCTACTTGCTTGAGAAGGCTCGCGTACATCAACAAGCTGGTAATGAACGAACTTTCCATATTTTCTACCAGTTACTTACTGGTGCAAATGATCAGTTGAAACGTGAACTTATGCTTGGTGACATCAGTAGTTACCGATTTATTTCAAACGGGATGCAGTCCATCACTGGACAAACTGATACCGAAGTTTTCCAAGAAACTTTGAGGGCGATGGAAATCATGAACATTTCCCACCAGGATGTTAAAGCTATGCTGAAAGTGGTCAGTGCAGTTTTATCTTTTGGCAACATGGAGTTTAAGAAGGAACGATCATCTGATCAAGCAGCCATGCCAGATAACACAGTCGCACA

>Myl9/12 in situ probe

GTAAAGAGGTTTCAGATGATTACATTGAGAAAATGTGTGGGGAAGCCCATGGTCCAATCAACTTTACCATGTTCCTTACAATGTTTGGCGAAAAGTTAAACGGCACTGACCCTGAGGAAGTTATAAGGAACGCCTTTGCTTGCTTTGATGAAGAAGGCACCGGAAAAATTAACGAGGATTATCTCCGAGAACTTCTCACTTGCATGGGTGATCGTTTCAATGATGATCAAGTGGATGAAATGTACAGAGAAGCTCCAATCAATGCTAAAACTGGTGACTTTGACTACATAGAGTTCACAAGGATTCTAAAGCATGGCGCTAAAGAAAAAGATGACATTTAGATACTATTAATAGTTGTATTCTACTGTCATCTGAATATATAATGAATCGATATTGGCCGATATAAGTACGATGGTCTGTCTTAAAACCTAGCACTCCTGAAATCAGTTTTTTTTTAAATAAAAACTGGTTGTTCTATTATTAAGGACTATGAAATGGTT

>Mylk in situ probe

TGAAAGATCCGCTTGGAAGTGGTCGATTCGGCAAAGTTTGGTTGGTGGAAGACCAGAAGACAGGTGACAAGTTCGCTGCCAAGCAATGCGCGTGTAGAAGGGCGAGCCAACGTAAGGAATTTGAGTTGGAGATTGAAATAATGAATTCACTCGATCATCCCAAGCTTCTACAACTCTACGACGCTTTCTTCGGGAAGAATGACGTCACACTTATTCTAGAATTGGTAACGGGGGGTGAATTATTCGATCGAATCGCTGACGAAGCGTTTGATCTAACGGAGGCGCTCGCTGTTAAATATATTCGACAAATATGTGAAGCTGTCCAGTACATGCACGGGAACATGATATTACATCTGGATATAAAGCCGGAGAACATATTATGCGTGTCACCTGAACGGTTGGACTCGATCAAAATCATTGACTTTGGGTTTGCACGCAAATACACCCCTAACTCACCCCTCAAAATAATGTTTGGAACCCCGGAGTTTGTTGCCCCCGAGGTCGTGAACTTTGACCCCCTGGGGAAGGGTACGGACATGTGGAGCATTGGGGTCGTGACCTATGTGCTATTATCAGGTTTGTCCCCTTTCATGGGTGAGGATGAACAGGAAACATTATCGAACGTAACGGCTTGCGATGTGGATTTTGATGATGATTCCTTCGATGACGTCAGTGATGATGCAAAGACATTTATTACTAAGCTGTTGGATATCAGGGAAAGCAAACGTCCCAACTGCGCTGAATGTTTGGAACACTCCTGGTTAAGCAAGGAAGATCAACACCGTAAAAGTTTGTCGGCTGCTGTTGTTAACCTCAAGAAGTTTGTGGCGCGGAGGAAATGGGTGAGATCGA

>Calponin in situ probe

ATGGCAAACCGACCAAAGGGATACGGATACTCTCGGGAAGTTGCTTCTAAGATTGATGCAAAGTACTCCGAAGAAGACGAAATGGAAGTCGTGGCTTGGGTCAGCTCCCTTGTTCAAAGTTCGCCGGAACAAGCAGGAAAAGAGGCTGGGATCTGTAAGCCCAACCCAACTTACACGGGCTCAATGCAGGCGATGAAACGGAACAAAGAGATGGAAAATATCGGAATGTTTCTTTCAGCAGCAGCAAAATACGGTGTCAAATCTGAGGACCAATTTCAAACGGTTGATTTGTACGAAGGTGGGAACTTGGGACAAGTTCAAGCCACTTTATATAAGCTTAGTTCAGTCGCCATGAAGAACGGAATGGGTGAAGGCATCGGTGTGAAAATAGCAGATGAAAACAAACGTAATTTTGACGAACAAAAAACGCGTGAAGGAAGAAACATCATTGGTTTACAGATGGGCACGAACCAAGTTGCTTCCCAAAGAGGCATGACAGCTTATGGACTCGGGCGACAATTGACTCCGCAAAAGTAG

>Atp2a in situ probe

TCCTGGATAGATGCACACATGTAAGGATTGGGAAGAACAAACATGAGATGACATCGGATATCAAGCAAAGTATATTGGAACGAGTGAAGGCATACGGAACTGGTCGTGATACTCTCAGATGTCTTGCTGTTGGTGTGGTTGATAACCCAATGAGCCCTAGTGATATGGATCTAAGTGATGCAACTAAATTTGCTAAATATGAGACTGGCATCACCTTCGTTGGAATCGTTGGAATGCTTGACCCACCTCGTCTTGAAGTATTTCAAGCTGTTCAGGATTGTAGAAACGCTGGAATTCGCGTCATCGTCATCACCGGTGATAACAAAGCAACTGCAGAAGCAATCTGCCGACGTATTGGTGTCTTCGGTGAAGATGAAGATACAACAGGTCGTTCATTCACTGGTCGTGAGTTTGATCAACTGAACCCAGAACAACAGTTCAAAGCTTGTCTTGAGGCTCGTCTTTTTGCTCGAGTGGAACCAGCTCACAAGAGCAAGATT

>Itpr in situ probe

TAGATGAGGACCAGAATCGTAAATTACAAGAATACCTCGCTACTACAGTCATGGATATCATCACCATGTTCTTTAATTCACCATTTTCAGATGCTACAACACTTATACAGACCCGTCAAACAACTTACATCCAATTGCTGCGTGCAATAGTGATGTTATACCAAAGTCCTTACCTACTGCCCCATTATAAACCTAAGCTTGAAGAAACCATTAAGAAGATGGGAGAAGTTGCTAAGAAGTGCAGCGTGGCGATCCCACAAGACGTGCAAAACCTCATCCTCCTACAGAGTAGCCTTAAGATGCTCGGTCAGCTAACACACTTGTTCTTACATCTGCATGTCCTGCATGCATTTGCCTGACACTGATTTTGTGATAGATTGTCTAAGCACGATTTTTCATTCTTAGTTTTCATTTCGTGTTTTTCAATTTACTAACAACAATTGACTCCGGTCTTGCCCTACATATCTGTTCATTCTAATTCTTTCTATTTTTCTACTGTC

>KH.C3.516 in situ probe

ATTGTAGGCGCATTTTAACAACATGGAAATCTCCGGCAACATTACAACAATGACAAATTTTCTGCTTACCAGTGACGTGTCGAGTGCGACTGAACTTAATGTGGCTCAAGGAAAAGTTCTAAGAGCGTCCGAGGACATGATGGTGGTTGCACTAATCGTGCTCAGTTTATACTTAACAGTAGCCTCCTTTGCTTACCAATGGAAGCAGCCGTCGCTCAGTGCCTTGAAAGGGACAAACAGATTATGCAGCGCCTGTGCAGTTATGCTTCTGGTCCAGTCGTTGTTTTTCGAAGTTGAAATGCGTGTCAGTTCTCCGACCTTACAGCTCTGTTATGCGTACTACGTAATCGTGCTAATTCTTGCCACAATCAACAAGTCACTAACATACTGTGTACTTTGGGTACGACAAAGGAGCTTTTACAAGAATCCTAAAAGTCAGTGTATGGTGTACTTCGGCAACGCAGTACTTACAGGAATCATCGTGCTGCCAATCTTACAGGTTGGGGTTTTAATGCTTAACATTGTTGACACGTCTCCCGTAGGGTGCATAACAAGAAAAGGGCCGGAGTTCTCGACAGTTTTAGTCCCGTTCGTATTTATGCTCTTCGTAGTTGTTCAACTGATTCTGCTTGGCCTCGTCCTCCGCCCAATTGTCCGCCATATCATGCGGAATAAGATCGGGCAAACTTCCAACTTGACGACGGTTGCAGTTCGTCTCAGCGTTTGCACAACACTCTGCGTTTTTACCGACCTTGCATTTTTGACAGTAGCGATAATTCAACCAAAGAACGTGGGATTTAGTTTTGGACCAATCGTCTTTCACTTTAACTCAACTGTCAATGTGGCAGCTATGCTGTGTTCGTTTGCTGATTACTCTGAACGTCTTATGCCGTTTTCACCATCCAACAACTCCAGTGTTTTAGAAGAGGATGGCCAGGAACCAAACACAGAGAAATCTCGAGTCTAAACACTTCAGATCCCCTAATACGCGAATTGTAATATTAATATCAACTTCCTTAAGTTTATAAACAGTTTTAAGTCCTGTTCGATTTGTTCCTAAAACTCACGTGTGTGCGAGTTACACTA

>Mef2 in situ probe

ATGGGTAGGAAGAAAATTCAAATTTCAAGAATCGGAGATGAACGTAACAGACAGGTCACGTTCACCAAGCGTAAGTTTGGTTTAATGAAGAAAGCTTATGAGTTGTCGGTGTTGTGCGACTGTGAGATCGCCCTCATCATATTCAACAGCTCCAATAAGTTGTTCCAATACGCAAGCACAGATATGGACAAGGTTTTGCTCAAATATACAGAATACAACGAACCACATGAAAGTAGAACCAATGCTGATATTATTGAGATGTTGAATAAGAAGGACAACAAGAACTGTGACAGCCCAGATGTCGACCACCAAGCTCAACTACCAACCCCTGGCACCTTGCAAAGATACGAAACAATCAACAAACAGTTTGACGACATGATTAACAGCATGAGGCCCCCCCCACAGCAGGACTTTTCATCAATGCCCGTCACGGTCCCAGTCACAGGAATTCACCCCCTACATGGTTACCCCTCTCCCATAGGAATGCACCAGATACAACATGAACAAGTCAACGACCCCAACCTACTTTACCCTCATCACCCACCCAACCGGCGCAGCCCCCTACCCAAGAGCCCCCTCGCACGTGGACACAGCCCCAGCCGGTCACCCTCAGGACAAATGTACAGCCCAGAGGTTAGCATGATATCTGATACACCTTCACCTGGAGGCAATGGGTACAGCGGGGGTAACCAGCCCCCGCCCCCCCAACAGCGGGGTCCATCACCCCACATGGATAGGACTTCCCCCATGCCCCACAAACCCATGCAACAAAGTCCCACTTCACCCGCACGGGGTCATCGACCGGGGTTGCATGTGGTGATTCCCCCATCGAGGAATATGCCAGGGATGGATGGGGGTAACGATCAACTGTCGACGCCGGTAGTTAGCCTCGCTACTCCGAATATCATCCAACAATATCAACACGCACCACAAACATCTTATAACCCGGTCGACTACCAAGTATTAGCGGGGGGTGAGGTCACGGGGTTCGCTTCCCCGGGGTCACTTCTGCAGAACTGGCCCCACCAATCCCCATTTCCCCAACCCCTAACCACCCCCCAACATCTACTGCCACACCCAAGTTTGATGACTCAGCAAGTCCCGATGATTCGAATAAAGCGCGAACCAGAGACGGAAGCTTCGCCCGACAAAGATCCGGATCAACGACCGGCTGCTGGAAGGTCACCCATGGAGGTGATGTCACCTTACGAGATGCGGATGGACGATCGTTCGATGATGGTGGAGAACGGTGGTAATCTTCCCGTGAAACGACAACGAGTTGAATCTGGTCCCAATGCCTCGCCCACGTGGACTACATGATGA

>Nk4 in situ probe

CGCACTCAAGACAAATCGAAACAAACAATATGGCTGCCTCCGATTCACAGTTCGACATGTTTTCGCAAAATGAAAACGCAAAATATTCTAAGGGGGCAATAGACACAAGGTATGCAGAAGCAACAAACACAGCAGACACGGCAGCGTCGAACCTTTTGCATTTTTCTCAAAATTTTCACCAAATTCCGTACGGGGAAAGTAGGGCACTGGTGGCGCCATTCGACATGCCTCGTTACGGTGGGGAGTATTTCAGTCCGACAAGCAACTACAGTGGAGGTTACACTCACCCCTATGACAACCCACCTAGCAATGTTGAAGCCTCACGATCGTCGTCGTTTCATTCACAGCAGTTTACGAAACCAAACGACGACGTTATTAGTACAACACACTCAACGTCCTCTGCCACAGCAGGAAACTTTTACCACAGACCGGCGAACAACTTCGAGCCTGTGAGAGAGCCAGGGCTGGAGAGATGTCCTGACAACGGAGACGAATTTTACACCAAAACTGAATCTCCGTTTCCCGCATTCAACCAACAGCATCAACCCACACCACAAAGCGAGCATCACAGCATGCCCAGCTACGACGAAATGAACGAGAAGATGTACACCGACCAGACATCCGAAACCGATATAACCAATAACTCAATGTTCGATTCGTCAAGTTCGGAGGCGGTGGTCGTCGATTTTCCATCACCATCCGGATCGCCAAACAAACAACATGAAGACGGAAGATTCGGAGGATCCTGTACGGATGAATCACTTCCGAGGACTTTCTCCGCCGCCGACATCCGGCAGGATGAATCAGTTACTCAAGTCACGGATTCGGAAAAGTCGGACAACGTCAGCACATGTAGCACTAGCCCCGAAGAAACTGAAAAAAAGAAAACAGAAGATGACACGCTAAAATCCCGACAC

>Foxg in situ probe

ATGACGAACGACGCGGCCGAGTCTGGTCATTCAAAGCGAGAAAACTTCATAGAGATGTCGCCCGAGTATTCAACGCTGATCGCCGCCGAAGATCGAAGCTCAGTTCCCCGCAGAGAGGACGCGCAAGTGCCGAGGTTTGAGAATATACAAAATGGTGGTGCCGATATAGAAAACGGTGAAGTGTCTCCAATACAAAAAGATATTGCAAACCAAGAGCTCAATGACGTAGCAATTAATATGACGTCACCTCAGAAACAAACAAACAACGAAAATTTAGAAGAAAAATGTCCAAAAGACCAGAAACCGTCAACAAGTCCACCGAGTAACAAGTACGGTAAGAAACCGCCATATTCATACAACGCCTTGATAATGATGGCCATCAAAAAGAGCCCACGAAAGCGACTTACACTAAGTCAGATCTACCAATACATAACAACTACATTCCCATACTACAAAGAAAATAAACAGGCGTGGCAGAATTCTATCAGACACAACTTATCGTTGAACAAATGCTTTGTGAAAGTACCGAGGCACTACGACGACCCCGGGAAGGGTAACTATTGGATGCTAGACCCGTCTAGTGATGATGTATACATTGGTAGTAGCACCGGTAAACTGAGACGAAGAAGTTCAAGCAGTCAAGCAAGGGGTCGTTTAGCATTACGACGAAGAACATTCGCCCAAGTATTTGGATCGCCGCAAGATATTTTACAACACGACCCCCCACAACAGATAATAAGAACAGACGTGACGTCACGAGCGGCCATGTTACGTCATCATGACGCAGCACGAACAATGCTGGGTTCTGGAATCCCACAGCGGGCAGATCCCTACCCTATGTTTCAACACACGCGGTTCCCTATGACTACAGAAACTGATTCAAGATACAGGCAATTGTATAGAGCGAGATTGGAACAATACTACGCGCATCTCGCGTCCTCTGCACTGTTCGGGCACATGCAAGCTACTGCATTAAACGCCCAGCCACGTATTGTTAAAAGCAACCCCGCCTCTCCTGAGCCTGTGCATTCCCATTACGAACAAACTACGTCACCAAACACCGCACCTCCTCGCTCTGACACCTCCACACCACCGCGAGGGATCGAACGACCGTGGGCATCACCGCCGCGTAGGAGGTGTGACGTCACAAAGCATGAAACAATTGACCGCGACGTCGTGTCGCGATGCTCCTCCGAATCTTCTAATGAATCATCAAGAAACAAGAGAGAAGTCACCGAAAACTCTACGAATTCACCGATACAGCAACTTAGTACAAGAGGAGGGTTACCGTTCTACCTTACACCAACCCCGAACCCTTGCCCTAATTTCTTAATGCCCAACACTGCCGGGTTAGTTCCAAATCCAAGTTACCCCTTCTTTTTCCCCCAACCATTCCACCCAGCCTTGGCGTTTCTCTCTCGGCCACAAACTGTTGCGTCATCATCGCAATTGTGA

>Myocardin in situ probe

TAGCCGAACAAGGCATAATTCCCCCCCTGAAAAGTCCCGGAACTTACTTTGAACAAGTTCAGCGCTTGCAGCGAGCAAAGAAGGAAGATTATCTTAACAGGGCAATTCAGATTCGGCCTGACAGAGACATGCTTGTACAAAAACATATTCTAGAAGACACAGTGGCTGCTGGATCAATTGTGGCAAGTCAGAAAGATTTAAAGAAAGCAAAGTTAGTTGATGATCTCAATGATAAAATTGCCTTCCGACCTGGGGTTATTGAACTAGTGGAGCGTAACATCTTCCCAGCCAATGACGACATACATGAAGCTATTAAAGGTGGTCATGTCCAGTATAAAAAGATCGCAGATTTTATGGAAGAGGATAGTAGTGATGCTCTTTCACCCGACCAACACCCCCCTCTTACATCAAGCCCACCTCTTATACTACAACTTGCCACCACAACCACTGCCATTGCATCTGTTGACAAAATTTCCTCCTTCCAGGCAAAAGCTGACCATAACTTTTCAAAAAGTAGCACAACGTCAATATTCACTCAGGGAAGCAGTCACCCATATCTTTCAAGACAAACATCGAAGGGTTCCAAGTTAAATCAAACCAATATTGGAATACCAAGAATATCAAATGCAAGAAGGAAAAAAGAAAAACCGAAAATGAAGAAATTCAAATACCATCAATATGTACCACCTGATATGCAAGGAAAAGACAAAGATCTTCCTAATCTCCAAGGAGATACACCTTATTCACGACTCTTACAGCAACAACAACTTTACCTTCAGTTTCAAATCATGAATAATCAACGTGCCGCACAACGGTTACCAATGGTCCCTCCTCCACCACCAGTGGTGCAATCCATGAAAATAGAGAAGCCACGGAAGACAAACACAGTTCTAACTCAGAACAAACTTGATGAAATGAAAGTTTCTGAGCTTAAGGAGGAACTGAAGGCACGTGGTTTGTCCGATCTTTGTTCTGACGAGAGTAACTCTGCATTCGACACTGCTGGCAGTTCGTGCATGCTGAATGATCTCTCCTACCCCAGCATGAATAAAGATGAACATTCAAACTCCACGCACACCACCAACAGTGAACACAATCC
